# A Digital Twin-Driven Sensing and Fuzzy Decision Framework for Safety Monitoring of Autonomous Mobile Robot Systems in Intralogistics

**DOI:** 10.3390/s26165284

**Published:** 2026-08-20

**Authors:** Sylwia Werbińska-Wojciechowska, Robert Giel, Olena Stryhunivska

**Affiliations:** 1 Faculty of Mechanical Engineering, Wroclaw University of Science and Technology, Wyspianskiego 27, 50-370 Wroclaw, Poland; sylwia.werbinska@pwr.edu.pl (S.W.-W.); robert.giel@pwr.edu.pl (R.G.); 2 Faculty of Management, AGH University of Krakow, 30-059 Krakow, Poland

**Keywords:** autonomous mobile robots, sensor-based monitoring, sensing systems, digital twin, fuzzy logic, fuzzy AHP

## Abstract

The increasing use of autonomous mobile robots (AMRs) in internal logistics systems improves operational efficiency. However, it also introduces challenges related to safety, reliability, sensor-based monitoring and human–robot interaction. This study proposes a sensor-driven Digital Twin and fuzzy decision-support framework for operational risk monitoring in AMR-based transportation systems. The proposed approach integrates Digital Twin technology with fuzzy logic methods to support continuous sensing, operational data acquisition, and data-driven risk evaluation in autonomous logistics environments. In the proposed framework, the Digital Twin acts as a continuous monitoring and early-warning environment. It enables continuous observation of system states, robot condition, navigation performance, traffic intensity and operational disturbances. To support decision-making under uncertainty, the fuzzy Analytic Hierarchy Process (fuzzy AHP) is applied to determine the relative importance of selected safety and reliability indicators. These indicators include condition monitoring parameters, mean time between failures, sensor-related disturbances and task completion performance. Subsequently, a hierarchical Mamdani fuzzy inference system is used to evaluate the operational risk level of the transportation system based on aggregated KPI values derived from Digital Twin data. The applicability of the proposed approach is illustrated through a case study involving multiple AMRs operating in a dynamic intralogistics environment. The results indicate that the integration of sensor-based Digital Twin monitoring with fuzzy decision-support mechanisms improves operational risk visibility and supports more effective risk identification and management in Industry 4.0 intralogistics systems.

## 1. Introduction

Internal transportation systems play a crucial role in modern industrial operations by enabling efficient material flow and streamlining logistics processes. They also improve coordination between different production stages [1]. In manufacturing plants, distribution centers, and warehouses, internal transportation systems support the movement of materials and goods between functional areas. With the development of Industry 4.0, autonomous mobile robots (AMRs) have become an important component of internal logistics, increasing operational flexibility and efficiency through autonomous navigation and transport task execution [2,3]. The implementation of mobile robots in internal transportation systems reduces labor costs, increases operational speed and improves the scalability, as well as flexibility, of logistics processes in dynamic environments [4,5]. Unlike traditional automated guided vehicles (AGVs), autonomous mobile robots (AMRs) rely on advanced sensing, mapping, and navigation technologies that enable autonomous decision-making and adaptive path planning in dynamic industrial environments. Although these capabilities enable operation in shared human–machine environments, they also introduce challenges related to safety, reliability and operational risk management [1,6].

System safety refers to a comprehensive approach aimed at ensuring that technical systems operate without causing unacceptable harm to people, property or the environment [7]. In internal transportation systems with mobile robots, maintaining a high level of safety is essential due to the potential consequences of failures, including production disruptions, material flow interruptions, equipment damage and risks to human workers. Navigation errors, mechanical malfunctions, communication losses and sensor faults may significantly affect both operational efficiency and system safety [8]. Therefore, sensor-based monitoring and early anomaly detection play an important role in improving operational safety and reliability in AMR transportation systems [9,10]. To address these challenges, organizations increasingly implement Safety Management Systems (SMSs) that integrate risk management, monitoring and preventive maintenance strategies. In mobile robot applications, such systems typically include continuous monitoring, diagnostics, predictive maintenance and risk analysis functions [11]. Risk management plays a key role in internal transportation systems by supporting the identification of hazards, evaluation of risks and implementation of mitigation strategies. Its importance is reinforced by dynamic operating conditions, frequent human–robot interactions and the need to maintain high system availability [12].

Despite the growing interest in safety and reliability issues related to autonomous mobile robots, several limitations remain in existing risk assessment approaches [13]. Traditional risk analysis methods often rely on deterministic assumptions and expert judgment, which may not adequately reflect the uncertainty and complexity of modern autonomous systems [13,14]. Many existing risk assessment approaches focus mainly on hazard identification and insufficiently integrate real-time operational and sensor-generated data into the evaluation process. As a result, the dynamic behavior of mobile robot systems and changing operating conditions are often inadequately reflected in risk models [15].

Unlike deterministic decision models that require precise numerical thresholds, fuzzy logic does not eliminate the need for expert knowledge. Instead, it formalizes expert reasoning through pairwise comparisons, linguistic variables, and fuzzy IF–THEN rules, while explicitly representing the uncertainty and imprecision inherent in expert assessments. Consequently, expert knowledge becomes a structured and transparent component of the decision-making process rather than an implicit source of subjectivity.

Another limitation concerns the fragmentation of existing approaches to safety and risk management in autonomous intralogistics systems. Previous studies have investigated Digital Twin technologies for real-time monitoring and anomaly detection, while other studies have applied fuzzy logic methods for safety and reliability assessment [10,16,17]. However, these techniques are often addressed separately or combined only partially, without providing a unified decision-support framework that simultaneously integrates continuous Digital Twin monitoring, KPI-based operational and safety assessment, fuzzy AHP weighting, and Mamdani fuzzy inference specifically for autonomous mobile robot transportation systems. Consequently, a research gap can be identified in the development of integrated frameworks capable of linking continuous Digital Twin-based monitoring with structured, uncertainty-aware risk evaluation under dynamic operating conditions. In particular, there is a need for approaches integrating sensor-based monitoring, Digital Twin environments, safety and reliability indicators and intelligent decision-support mechanisms in autonomous intralogistics systems. To address this gap, this study proposes an integrated risk management framework that combines Digital Twin-based monitoring with fuzzy logic-based risk assessment for mobile robot transportation systems. The proposed approach integrates continuous operational monitoring, fuzzy AHP for weighting risk indicators and a Mamdani fuzzy inference system for aggregated risk evaluation, supporting more effective risk identification and management in dynamic industrial environments.

The main contribution of this study is the development of a unified decision-support framework for autonomous mobile robot transportation systems that integrates Digital Twin-based monitoring with fuzzy logic-based risk assessment. In contrast to existing approaches, the proposed framework combines continuous sensor-driven monitoring, KPI-based safety evaluation, fuzzy AHP weighting, and Mamdani fuzzy inference into a single coherent structure for dynamic risk assessment in intralogistics environments. To further position the proposed framework with respect to existing research, a comparative analysis of related Digital Twin- and fuzzy logic-based risk assessment studies is presented in Section 2. To address the identified research gaps, the following research questions were formulated: 

RQ1. How can Digital Twin-based monitoring be integrated with fuzzy logic-based risk assessment to improve safety and reliability in AMR-based intralogistics systems?

RQ2. Which key performance indicators (KPIs) most effectively reflect the operational and safety performance of AMR-based logistics systems?

RQ3. How does the proposed framework compare with traditional risk assessment methods in terms of uncertainty handling and decision-support capability?

RQ4. Can the proposed framework provide robust decision support for maintenance planning, safety investments and operational optimization under uncertainty?

In line with these research questions, this study aims to achieve the following objectives:

- To design an integrated risk management framework for mobile robot internal logistics systems, combining Digital Twin monitoring and fuzzy logic risk assessment;
- To identify and prioritize key performance indicators relevant for system safety and reliability evaluation using the fuzzy AHP;
- To develop a Mamdani fuzzy inference system for quantifying overall risk levels based on selected KPIs;
- To demonstrate the applicability and robustness of the proposed framework using a representative industrial-inspired case study based on operational data from an AMR development project.

The remainder of this paper is organized as follows. Section 2 reviews the literature on safety and risk management in mobile robot transportation systems, Digital Twin applications, sensor-based monitoring and fuzzy logic-based risk assessment methods. Section 3 presents the proposed integrated risk management framework, while Section 4 describes the fuzzy logic-based methodology, including the fuzzy AHP and the Mamdani inference model. Section 5 presents the case study and application of the proposed approach. The proposed framework is demonstrated using representative operational data collected during a collaborative research project focused on the development of autonomous mobile robots for internal logistics, combining experimental monitoring data with Digital Twin simulation environments. Section 6 discusses the results and their implications for safety and reliability management. Finally, Section 7 concludes this paper and outlines directions for future research.

## 2. Risk Management and Sensor-Based Monitoring in Mobile Robot Transportation Systems–Literature Review

### 2.1. Internal Logistics Systems with Mobile Robots and Sensor-Based Monitoring

Internal logistics systems are responsible for the movement, storage and handling of materials within industrial facilities, warehouses and distribution centers [18]. Increasing operational complexity and the growing demand for flexibility have accelerated the adoption of mobile robotic systems, particularly automated guided vehicles (AGVs) and autonomous mobile robots (AMRs), supported by sensor-based monitoring technologies [18,19]. Unlike AGVs, which typically operate along predefined routes, AMRs employ advanced sensing, mapping, and navigation technologies, including LiDAR, cameras, proximity sensors and simultaneous localization and mapping (SLAM), to enable autonomous operation in dynamic industrial environments [20,21]. Although these capabilities improve operational flexibility and efficiency, they also increase system complexity, uncertainty, and safety challenges, highlighting the need for continuous monitoring and advanced risk management. To illustrate the differences between robotic transportation technologies and their implications for safety and risk management, Table 1 compares AGVs, AMRs, and warehouse robotics systems with respect to their navigation, sensing, operational characteristics, and associated risk management requirements. The comparison demonstrates that increasing levels of autonomy and sensing capabilities improve operational flexibility but simultaneously require more sophisticated monitoring and risk assessment mechanisms.

Among the available robotic transportation technologies, AMRs have become increasingly important in modern intralogistics due to their ability to operate autonomously in dynamic environments and collaborate with human workers [22,23]. They are widely applied in goods transportation, order picking, inventory handling, and material delivery, often as part of integrated warehouse management systems (WMSs) or manufacturing execution systems (MESs) [24]. These applications rely on continuous streams of operational and sensor data describing robot status, navigation performance and interactions with the surrounding environment.

Despite their operational advantages, AMR-based transportation systems introduce new challenges related to safety, reliability and operational risk. Dynamic interactions among autonomous robots, human operators and technical infrastructure generate uncertainty that cannot be adequately addressed using conventional static risk assessment approaches [15,23,25]. Consequently, ensuring safe and reliable operation requires continuous sensor-based monitoring combined with decision-support methods capable of processing dynamic operational information and identifying emerging risks in real time. These requirements have stimulated growing interest in Digital Twin technology as a platform for integrating real-time monitoring with safety and risk management in Industry 4.0 intralogistics systems.

### 2.2. Safety and Sensor-Based Monitoring Challenges in Autonomous Transportation Systems

Safe and reliable operation is a fundamental requirement for autonomous transportation systems deployed in modern intralogistics. In AMR-based environments, safety and reliability are closely interconnected, as failures in sensing, localization, or communication may simultaneously reduce operational continuity and increase the likelihood of hazardous situations [9,13]. Consequently, continuous sensor-based monitoring and real-time operational data acquisition have become essential for supporting navigation, obstacle detection and early anomaly identification in dynamic industrial environments.

In addition to safety and reliability, several complementary concepts are commonly considered when evaluating the operational performance of autonomous logistics systems. These include availability, resilience, and operational efficiency, all of which contribute to the overall capability of the system to maintain stable and safe operation under varying conditions. Table 2 summarizes the principal system performance concepts and highlights their relevance to autonomous mobile robot logistics systems.

Although these concepts describe different aspects of system performance, they are strongly interrelated in autonomous transportation systems. Safety focuses on preventing hazardous events; reliability and availability describe operational continuity, whereas resilience reflects the capability of the system to withstand and recover from unexpected disturbances. All of these aspects increasingly depend on the quality of sensor-based monitoring and the timely interpretation of operational data.

Recent studies indicate that navigation reliability remains one of the primary challenges in AMR-based logistics systems [24,27,28,29]. Autonomous mobile robots rely on the continuous integration of data from LiDAR sensors, cameras, proximity sensors and inertial measurement units to support localization, environmental mapping and obstacle detection. Errors in sensor measurements, localization algorithms or environmental perception may lead to route deviations, reduced navigation accuracy and an increased risk of collisions, particularly in environments shared with human operators [28,29].

Another important challenge concerns the reliability of sensing and communication systems. Sensor degradation, calibration errors, environmental interference or communication failures may significantly reduce the accuracy of environmental perception and coordination between autonomous robots and warehouse management systems [11]. Since AMRs frequently operate in collaborative human–robot environments, delayed obstacle detection or failures in human presence recognition may directly affect operational safety [30]. Furthermore, communication disruptions or software integration failures may result in fleet coordination errors, transport delays and temporary loss of system visibility, thereby reducing both operational efficiency and system reliability [24].

Table 3 summarizes the principal categories of safety and reliability challenges reported in the literature, together with their typical causes and operational consequences. The overview demonstrates that many operational risks originate from sensing, communication, and environmental uncertainties rather than from purely mechanical failures.

The diversity of risk sources highlights the limitations of conventional deterministic risk assessment approaches, which often rely on static assumptions and are not well-suited to continuously changing operating conditions. In autonomous transportation systems, operational information is inherently uncertain due to sensor inaccuracies, environmental variability, and the dynamic interaction between robots and human operators. Consequently, there is growing interest in intelligent decision-support methods capable of integrating heterogeneous operational data while explicitly accounting for uncertainty. Among these approaches, fuzzy logic has emerged as an effective technique for supporting safety assessment and risk evaluation in complex industrial systems. The following section, therefore, reviews recent fuzzy logic-based methods applicable to autonomous transportation and industrial safety management.

### 2.3. Risk Assessment and Sensor-Based Monitoring Methods in Autonomous Logistics Systems

Risk assessment plays a crucial role in ensuring the safe and reliable operation of autonomous logistics systems. In AMR-based environments, increasing system autonomy, continuous sensor-based monitoring and real-time operational data acquisition have significantly changed the way risks are identified and evaluated [1,31]. Consequently, risk assessment methods are expected not only to identify hazards but also to support continuous decision-making based on dynamically changing operational conditions.

Traditional approaches to risk assessment remain the foundation of safety management in industrial systems. ISO 31000 provides a general framework for systematic risk identification, analysis and evaluation, but focuses primarily on procedural aspects rather than the integration of operational monitoring data into the assessment process [32]. Engineering methods, such as Failure Mode and Effects Analysis (FMEA) and Fault Tree Analysis (FTA), are widely applied to identify failure mechanisms and analyze causal relationships in complex technical systems [33,34,35]. In the context of autonomous logistics, these methods have been successfully applied to evaluate failures related to navigation, sensing and communication systems [33]. Probabilistic approaches, including Probabilistic Risk Assessment (PRA) and Bayesian networks, further extend traditional analyses by enabling quantitative estimation of failure probabilities and dynamic updating of risk estimates based on new observations [36,37]. Nevertheless, these approaches often require extensive historical datasets, well-defined probabilistic distributions and assumptions that may be difficult to satisfy in highly dynamic autonomous logistics environments [1].

To overcome these limitations, recent research has increasingly focused on uncertainty-aware risk assessment methods. In particular, fuzzy extensions of classical engineering techniques have demonstrated improved capability to incorporate imprecise information and expert knowledge into the evaluation process. Fuzzy FMEA has been successfully applied to warehouse operations and autonomous logistics systems, providing more flexible prioritization of failure modes than conventional deterministic scoring methods [33,34,38]. Similarly, hybrid approaches integrating FTA with multi-criteria decision-making techniques, such as the CM-improved AHP proposed by Zheng et al. [39], enhance the evaluation of safety risks by combining causal analysis with structured weighting of risk factors.

Beyond extensions of traditional methods, fuzzy logic has become one of the most widely adopted approaches for decision support in complex engineering systems characterized by uncertainty. Fuzzy inference systems enable the representation of linguistic knowledge and uncertain relationships among operational variables, supporting risk evaluation when sensor measurements are incomplete, noisy or difficult to interpret [40,41]. Recent studies have successfully applied fuzzy logic to autonomous vehicles [42], automotive safety assessment [43], supply chain risk aggregation [44] and autonomous decision-support systems processing large-scale operational data [45]. These studies demonstrate that fuzzy approaches provide a flexible framework for integrating heterogeneous information sources while supporting transparent and interpretable decision-making under uncertainty.

At the same time, advances in autonomous logistics have significantly increased the availability of real-time operational information through sensor networks, IoT infrastructures and robot monitoring systems [1,46]. Continuous streams of data describing robot localization, navigation performance, battery condition, communication quality and environmental interactions create new opportunities for dynamic risk assessment. However, transforming these heterogeneous data into meaningful risk indicators remains a major challenge. Existing studies frequently employ sensor-based monitoring to detect anomalies or evaluate system performance, whereas the integration of continuous operational monitoring with intelligent risk evaluation remains comparatively limited.

Table 4 summarizes the principal risk assessment methods reported in the literature together with their main characteristics, advantages and limitations in the context of autonomous logistics systems.

The reviewed literature demonstrates a clear evolution from deterministic engineering methods toward intelligent, uncertainty-aware decision-support approaches. Classical techniques, such as FMEA and FTA, and probabilistic models remain valuable for systematic hazard identification and reliability analysis; however, they provide limited capability for processing uncertain, heterogeneous and continuously changing operational data. Fuzzy logic-based methods address many of these limitations by combining expert knowledge with sensor-generated information and supporting flexible risk evaluation under uncertainty. Nevertheless, existing studies remain largely fragmented. Classical engineering methods rarely exploit real-time monitoring data, while fuzzy decision-support models are typically developed independently of Digital Twin environments. Consequently, there remains a need for integrated frameworks capable of combining continuous sensor-based monitoring, structured KPI evaluation and fuzzy inference within a unified Digital Twin-enabled decision-support architecture for autonomous logistics systems.

### 2.4. Digital Twin Application and Sensor-Based Monitoring in Logistics and Transportation Systems

The development of Industry 4.0 has significantly increased interest in Digital Twin technology as a key enabling paradigm for monitoring, analyzing, and optimizing complex industrial systems [47]. In general terms, Digital Twins integrate sensor-generated data, simulation models, and analytical tools to enable real-time monitoring, anomaly detection, and operational decision-making in logistics environments [48,49]. However, in the context of autonomous logistics systems, Digital Twin technology should be understood not only as a monitoring and simulation environment, but also as a data-driven decision-support layer enabling continuous interpretation of sensor-generated information under uncertainty [50,51].

In logistics and transportation systems, Digital Twins are primarily implemented across several functional domains. A substantial body of the literature focuses on real-time monitoring of system states, including vehicle position, operational status, and resource utilization, supported by IoT and sensor data integration [47,52]. Another widely explored area is operational performance analysis, where Digital Twins are used to evaluate efficiency indicators, such as throughput, congestion levels, and task execution rates in warehouse and intralogistics systems [53,54]. Predictive maintenance represents another dominant application, where sensor data from robotic platforms, including AMRs and AGVs, are processed to detect early signs of degradation in mechanical, electrical, and navigation subsystems [16,55,56]. In addition, simulation-based scenario analysis enables evaluation of alternative logistics configurations, including routing strategies, warehouse layouts, and fleet management policies without disrupting real operations [57,58].

Recent studies extend Digital Twin applications toward more advanced decision-support functionalities. In particular, risk monitoring and early-warning systems have been proposed to detect abnormal operational states based on deviations between real-time sensor data and expected system behavior within the digital replica [59,60]. Such approaches are increasingly relevant in autonomous logistics environments, where system complexity, human–robot interaction, and dynamic operating conditions introduce significant uncertainty. However, most existing solutions remain focused on anomaly detection or performance monitoring rather than comprehensive risk evaluation frameworks that explicitly quantify and prioritize risk factors.

More recent research has begun to integrate Digital Twins with risk assessment and safety management methodologies. Zio and Miquelés [16] highlight the potential of Digital Twins in safety analysis and risk-informed decision-making, particularly in systems requiring continuous monitoring and dynamic response capabilities. Similarly, Li and Wang [61] propose Digital Twin-driven management strategies for logistics systems, emphasizing optimization and operational control, although without explicit modeling of uncertainty in risk evaluation. In the context of safety-critical applications, Virando et al. [62] demonstrate how Digital Twin simulation can support safety risk management processes by enabling scenario testing and hazard identification in complex systems. Kampourakis et al. [63] further extend this perspective by reviewing Digital Twin-enabled incident detection and response mechanisms in critical infrastructure systems, highlighting their role in early-warning and resilience enhancement.

In parallel, artificial intelligence and data-driven approaches are increasingly being integrated with Digital Twin environments to enhance predictive and adaptive capabilities. Sajadieh and Noh [64] show that combining AI techniques with Digital Twins enables the transition from simulation-based systems toward autonomous decision-support environments. Similarly, AI-enhanced Digital Twin frameworks for warehouse logistics have been proposed to improve optimization, adaptability, and operational intelligence in complex logistics systems [65]. From a broader perspective, data-enabling technologies and AI-driven architectures are identified as key enablers of next-generation Digital Twin systems across industrial applications [66].

Despite these advancements, most existing Digital Twin-based solutions in logistics and transportation remain primarily focused on monitoring, simulation, and optimization tasks. Risk-related functionalities are typically limited to anomaly detection or qualitative safety assessment, without formal integration of structured risk evaluation models. Moreover, uncertainty in sensor data, environmental variability, and human–robot interaction is often addressed using deterministic thresholds or rule-based logic, which may not adequately capture the complexity of real-world autonomous logistics systems. To address these limitations, recent studies have begun exploring the integration of Digital Twins with advanced decision-support methodologies. Okimi et al. [67] propose a framework combining Digital Twins with decision-support systems for predictive risk management, demonstrating improved risk awareness in dynamic environments. However, the approach remains domain-specific and does not explicitly address fuzzy uncertainty modeling. Similarly, Li et al. [59] analyze Digital Twin-enabled risk management capabilities in logistics systems from an innovation diffusion perspective, emphasizing organizational adoption rather than methodological integration. Galkin et al. [68] provide a bibliometric analysis of Digital Twin applications in logistics, confirming the rapid growth of research in this area but also identifying a lack of unified methodological frameworks for risk assessment.

To provide a structured overview of existing approaches and to identify the main research gaps, Table 5 summarizes selected Digital Twin applications in logistics and transportation systems, with particular emphasis on sensing capabilities, real-time monitoring, and decision-support functions.

Collectively, the analyses presented in Table 2, Table 3, Table 4 and Table 5 demonstrate that, although substantial progress has been achieved in the areas of safety management, risk assessment, and Digital Twin technologies, an integrated framework combining continuous sensor-based monitoring with hierarchical, multi-level, and uncertainty-aware risk evaluation for AMR-based intralogistics systems has not yet been reported in the literature. Additionally, despite the significant progress in Digital Twin applications for logistics systems, the analysis of the literature reveals a clear research gap in the area of integrated risk-aware Digital Twin frameworks. Existing studies predominantly treat Digital Twins as tools for visualization, simulation, or performance monitoring, while risk assessment is typically addressed separately using conventional reliability or safety engineering methods. As a result, there is a lack of unified approaches that combine continuous sensor-based monitoring with intelligent risk evaluation capable of handling uncertainty and dynamic system behavior.

Another important limitation is that most existing Digital Twin-based risk-related approaches do not explicitly incorporate multi-level system perspectives. In particular, the interdependencies between robot-level condition monitoring, interaction-level safety dynamics, and fleet-level coordination processes are rarely modeled within a single coherent framework. This limits the ability of current approaches to capture emergent risk phenomena in autonomous logistics systems.

To address these limitations, there is a need for integrated frameworks that combine Digital Twin-based real-time monitoring with advanced uncertainty-aware risk modeling techniques. In particular, fuzzy logic-based approaches provide a promising direction for bridging this gap, as they enable the representation of linguistic knowledge, partial truth, and imprecision inherent in sensor-generated data and expert assessments. Such integration allows for more realistic modeling of operational conditions in autonomous logistics environments and supports proactive decision-making under uncertainty.

Building upon these observations, this study proposes an integrated Digital Twin-based decision-support framework that bridges the identified methodological gaps. Unlike previous approaches, the proposed framework combines continuous sensor-based monitoring, hierarchical KPI modeling, fuzzy AHP weighting, and Mamdani fuzzy inference within a unified architecture dedicated to AMR-based intralogistics systems. Consequently, the framework supports not only real-time system observation but also structured, uncertainty-aware risk interpretation across the robot, interaction, and fleet levels. The conceptual architecture and methodological details are presented in Section 3.

## 3. Integrated Sensor-Based Monitoring and Risk Management Framework for Mobile Robot Logistics Systems

### 3.1. The Concept of the Integrated Sensor-Based Monitoring and Risk Management Framework

**Figure 1:**
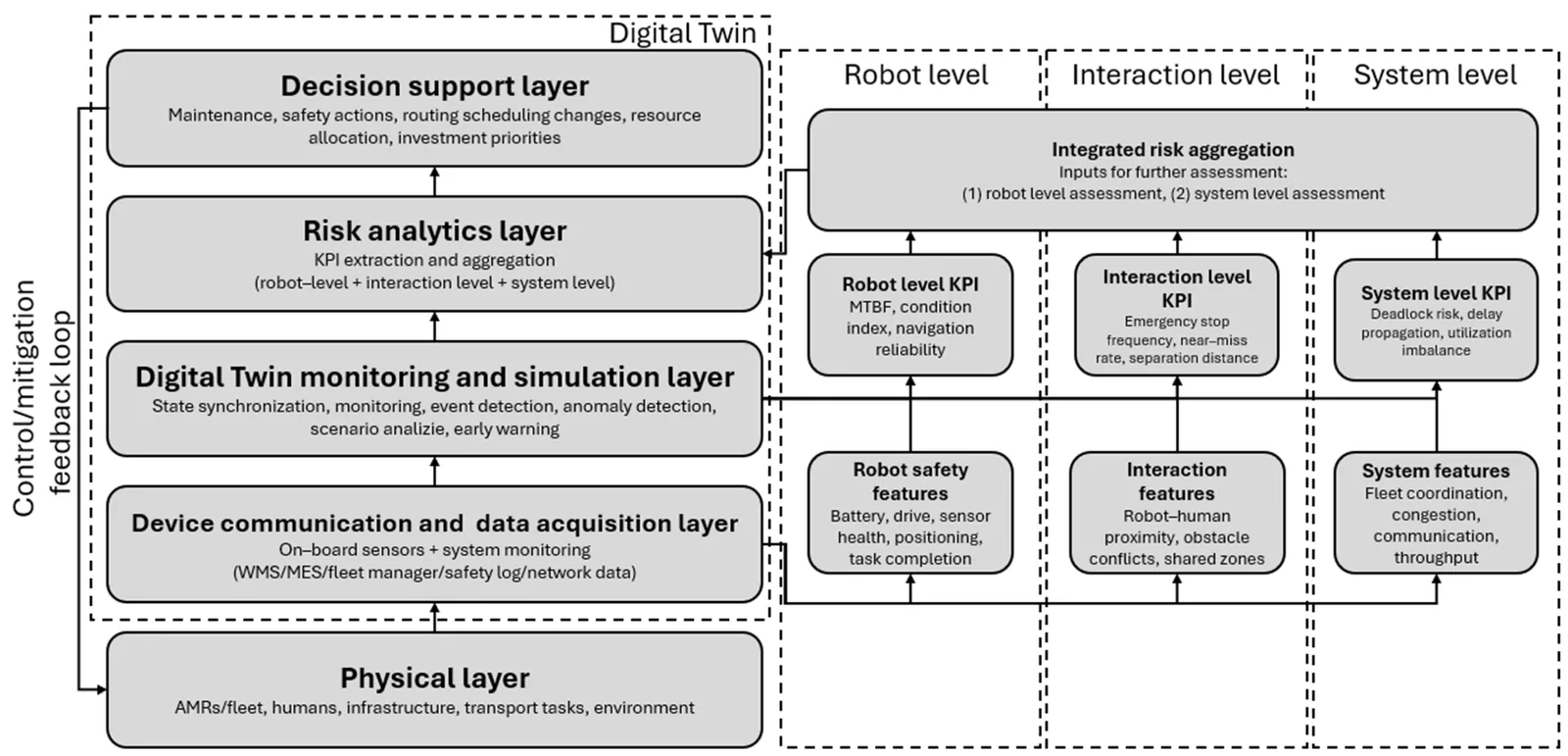
Integrated sensor-based monitoring and risk management framework for mobile robot logistics systems. Source: Own contributions, based on [73,74,75,76].

Risk assessment in AMR-based intralogistics systems should not be limited to isolated technical failures of individual robots. The overall risk profile results from interactions among robot conditions, robot–environment, and robot–human interactions, as well as fleet-level coordination processes. Therefore, the proposed framework adopts a hierarchical perspective integrating three analytical levels: the robot, interaction and fleet/system level. This approach combines local operational signals with supervisory information. As a result, it provides a more comprehensive representation of safety- and reliability-related risks in dynamic logistics environments. The framework combines two complementary reference points. It is conceptually aligned with ISO 31000 [32] at the level of risk management logic and inspired by ISO 23247 at the level of Digital Twin architecture and data flows [73,74,75,76]. While ISO 31000 defines the general structure for risk management, ISO 23247 supports the integration of physical systems, communication layers, digital representations and decision-support processes within a coherent Digital Twin environment. The proposed framework extends this perspective to multi-robot intralogistics systems by integrating sensor-based monitoring, KPI aggregation and intelligent risk evaluation within a unified structure. In this approach, the Digital Twin acts not only as a monitoring environment, but also as a basis for extracting risk-related information and supporting safety-oriented decision-making in autonomous logistics systems. Furthermore, the framework supports the integration of sensor-generated monitoring data with intelligent risk evaluation mechanisms. Additionally, the proposed framework separates the continuous monitoring layer from the decision layer. While the Digital Twin continuously acquires operational data, the fuzzy inference engine operates on KPI values computed over configurable observation windows, whose duration may be adjusted depending on the required responsiveness of the application. The resulting concept is shown in Figure 1.

In the proposed framework, risk is analyzed across three complementary levels: the robot, interaction, and fleet/system level. The robot level focuses on the condition and performance of individual AMRs, the interaction level considers relationships between robots and their environment, while the fleet/system level reflects coordination and overall operational performance of the logistics system. This multi-level structure supports integrated sensing, monitoring and interpretation of operational data. The physical layer represents the real operational environment, including AMRs, robot fleets, human operators, infrastructure and transport tasks. It integrates sensing systems, monitoring devices and operational data acquisition mechanisms used to continuously observe system conditions and robot behavior in dynamic logistics environments. The data acquisition and communication layer is responsible for collecting and transmitting operational information from robot sensors, WMS, MES, fleet management systems and communication networks. Through sensor fusion and continuous data synchronization, this layer improves system visibility and supports anomaly detection.

The Digital Twin layer provides a synchronized digital representation of the physical system and its current operational state. It supports continuous monitoring, event detection, and anomaly identification, as well as scenario analysis by transforming raw operational data into structured information describing robot condition, interaction states, and system behavior. The risk analytics layer extracts and aggregates risk-related indicators at the robot, interaction and fleet/system level. By integrating sensor-generated operational data with risk evaluation mechanisms, it provides a comprehensive analytical basis for identifying local disturbances and system-wide operational risks.

The subsequent risk evaluation combines objective operational data obtained from the Digital Twin with structured expert knowledge used to define KPI priorities, linguistic variables and fuzzy inference rules. In this way, expert knowledge complements sensor-derived information rather than replacing it, allowing uncertainty in engineering judgment to be explicitly represented within the decision-making process.

Moreover, in the article, a separate section/comment concerning the role of the Digital Twin in sensor-based monitoring of internal logistics systems has been introduced. The Digital Twin is included in the proposed framework because risk assessment in AMR-based internal logistics systems should rely on continuously updated information about the actual operating state of the system rather than only on predefined hazards or static expert judgments. In dynamic logistics environments, operating conditions may change due to robot failures, navigation deviations, sensor disturbances, communication problems or interactions with humans and infrastructure. Therefore, continuous sensor-based monitoring and real-time operational data acquisition are essential for detecting anomalies and maintaining reliable system observation.

In the proposed framework, the Digital Twin acts primarily as a monitoring and early-warning environment. Its role is not to perform the final risk evaluation, but to provide a synchronized and interpretable representation of the current system state. By integrating real-time operational data, the Digital Twin supports continuous observation of robot condition, operational behavior, and environmental factors, while enabling early detection of abnormal states and operational deviations. This approach is consistent with previous studies identifying Digital Twins as effective tools for monitoring, diagnosis, simulation and maintenance-oriented decision support in internal transportation systems [56]. An important advantage of the proposed Digital Twin framework is its ability to integrate data from multiple analytical levels, including the robot, interaction and fleet/system level. As a result, the Digital Twin transforms raw operational and sensor-generated data into structured information describing system condition, deviations and warning patterns. These outputs subsequently support the identification of key performance indicators used in intelligent risk evaluation and safety-oriented decision-making in autonomous logistics systems.

### 3.2. Identification of Sensor-Based Key Performance Indicators for Risk Evaluation

The Digital Twin environment provides the operational data required to quantify the selected KPIs, whereas the selection and hierarchical organization of the indicators were established based on the literature analysis and expert knowledge from specialists in autonomous logistics systems. These data are continuously acquired through the Digital Twin monitoring infrastructure, integrating onboard sensors and system-level data sources. In the proposed framework, the indicators are organized according to three analytical levels: the robot level, interaction level and fleet or system level. The indicators additionally integrate sensing-related information and monitoring data obtained from autonomous transportation systems. Table 6 presents a preliminary set of KPIs that may serve as candidate inputs for the subsequent fuzzy logic-based assessment model. Particular attention is given to sensor-based operational indicators and monitoring-related parameters supporting real-time risk evaluation.

Some of the proposed indicators represent directly measurable performance variables, whereas others are composite indicators derived from operational signals available in the Digital Twin environment. These signals are continuously acquired through the Digital Twin data acquisition layer, integrating onboard sensing systems and fleet-level operational logs. This structure enables the integration of technical, operational and safety-related aspects within a unified multi-level risk assessment framework. It also supports intelligent risk evaluation through the analysis of sensor-generated monitoring and operational data in autonomous logistics systems.

The final set of KPIs is selected considering both the literature evidence and engineering relevance for AMR-based intralogistics systems. Expert knowledge is subsequently employed during the weighting process and in the formulation of the fuzzy inference model described in Section 4.

## 4. Fuzzy Logic-Based Risk Assessment Method

### 4.1. Selection of Risk Indicators Using Fuzzy AHP

The key performance indicators identified in Section 3 represent different aspects of AMR-based logistics system behavior, including robot condition, robot–environment interactions and fleet coordination performance. However, their contribution to the overall risk level may differ depending on their influence on safety, reliability and operational continuity. Therefore, a structured weighting approach was required before incorporating these indicators into the fuzzy risk assessment model. To address this issue, the weighting process was performed using the fuzzy Analytic Hierarchy Process (fuzzy AHP). This method extends the classical AHP by incorporating fuzzy set theory to represent uncertainty in expert judgments [77,78]. In autonomous logistics systems, the relative importance of indicators is often expressed using linguistic evaluations rather than precise numerical values, making fuzzy numbers suitable for risk evaluation. The first step involved defining a hierarchical decision structure for operational risk evaluation in the AMR-based logistics system. The hierarchy consists of three analytical levels introduced in Section 3: the robot level, interaction level and fleet/system level. The final level includes the specific key performance indicators identified within the Digital Twin environment.

In the proposed framework, expert knowledge is used to capture qualitative engineering experience that cannot be directly inferred from operational data. Specifically, experts contribute to the pairwise comparison of KPI importance within the fuzzy AHP procedure. Their judgments concern the relative significance of indicators for operational safety and system reliability rather than the assessment of individual operational events. Consequently, expert knowledge is employed only to establish the structural weighting of the model, whereas the subsequent risk evaluation is performed automatically using operational data acquired from the Digital Twin environment.

Pairwise comparisons between indicators were performed using linguistic judgments provided by domain experts with experience in autonomous intralogistics systems. Each linguistic term was represented by a triangular fuzzy number (TFN) defined as:

(1) $$\tilde{A}=\left(l,m,u\right)$$

where *l*, *m*, and *u* represent the lower, modal, and upper values of the fuzzy number, respectively.

The use of linguistic assessments reflects the fact that the relative importance of safety indicators cannot usually be expressed precisely using deterministic numerical values. Instead, experts evaluate whether one indicator is moderately, strongly or very strongly more important than another with respect to the overall system risk. These qualitative assessments are subsequently transformed into triangular fuzzy numbers, according to the scale presented in Table 7.

Based on this scale, fuzzy pairwise comparison matrices were constructed for each group of indicators. The elements of the matrix are represented by triangular fuzzy numbers describing the relative importance of criterion *i* with respect to criterion *j*:

(2) $$\tilde{A_{ij}}=\left(l_{ij},m_{ij},u_{ij}\right)$$

The fuzzy synthetic extent value for each criterion was then calculated using the extent analysis method. For criterion *i*, the fuzzy synthetic extent value $S_{i}$ is determined as:

(3) $$S_{i}=\left(\sum_{j=1}^{m}M_{ij}\right)⨂{\left(\sum_{i=1}^{n}\sum_{j=1}^{m}M_{ij}\right)}^{-1}$$

where $M_{ij}$ represents the triangular fuzzy comparison values and the operator ⊗ denotes fuzzy multiplication.

Next, the degree of possibility that one fuzzy number is greater than another was calculated in order to determine the relative dominance of the criteria. The degree of possibility of $S_{i}\geq S_{j}$ is expressed as:

(4) $$V\left(S_{i}\geq S_{j}\right)=\left\{\begin{array}{c} 1,\;\;\;\;\;\;\;\;\;\;\;\;\;\;\;\;\;\;\;\;\;if\;\;m_{i}\geq m_{j}\\ 0,\;\;\;\;\;\;\;\;\;\;\;\;\;\;\;\;\;\;\;\;\;if\;\;\;\;l_{j}\geq u_{i}\\ \frac{l_{j}-u_{i}}{\left(m_{i}-u_{i}\right)-\left(m_{j}-l_{j}\right)},\;otherwise \end{array}\right.$$

Based on these values, the normalized weight vector for the criteria was obtained. Finally, the fuzzy weights were defuzzified to obtain crisp priority values representing the relative importance of the indicators. In this study, defuzzification was performed using the centroid method:

(5) $$w_{i}=\frac{l_{i}+\;m_{i}+u_{i}}{3}$$

The resulting normalized weights represent the relative importance of the indicators and are subsequently used as parameters in the fuzzy inference models developed in the next section. Since the expert judgments affect only the weighting coefficients, their influence on the final risk assessment is indirect. The final risk value additionally depends on real operational KPI values, the fuzzy membership functions and the inference rule base. Consequently, no single expert judgment can determine the final risk level independently. By integrating the fuzzy AHP weighting procedure with the multi-level Digital Twin-based monitoring framework, the proposed approach ensures that the fuzzy risk assessment model reflects both the structural relationships between indicators and their relative contribution to system risk.

### 4.2. The Development of the Mamdani Fuzzy Inference Model

**Figure 2:**
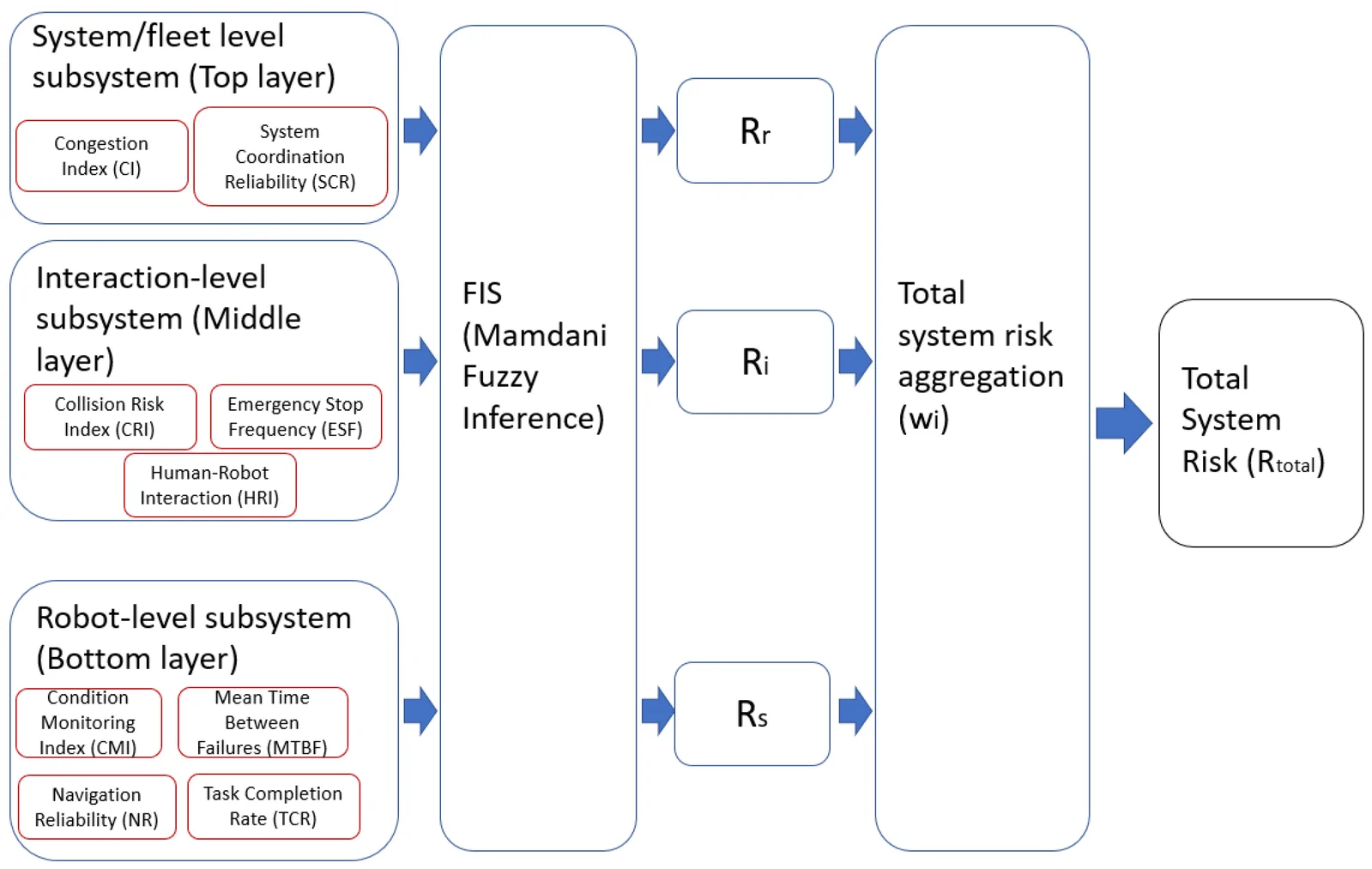
The architecture of the hierarchical fuzzy risk model.

The proposed risk assessment model was developed as a hierarchical fuzzy inference system reflecting the multi-level structure of the internal logistics system introduced in Section 3. In autonomous transportation environments, safety and reliability are influenced by factors related to robot condition, robot–environment interactions and fleet coordination. Therefore, the framework was designed as a multi-layer structure in which different categories of operational indicators are evaluated separately and then integrated into a single system-level risk indicator. The fuzzy risk evaluation model consists of three interconnected inference modules corresponding to the robot, interaction and fleet/system levels (Figure 2). Each module processes operational indicators representing a specific dimension of system performance. The indicators, identified in Section 3.2 are obtained from the Digital Twin monitoring environment, which continuously collects operational data related to robot condition, safety interactions and coordination performance.

At the robot level, the fuzzy subsystem evaluates risks related to the technical reliability and operational performance of individual autonomous mobile robots. The input variables include the Condition Monitoring Index, Mean Time Between Failures (MTBF), Navigation Reliability and Task Completion Rate. Based on these indicators, the subsystem generates the robot-level risk indicator $R_{r}$, representing the reliability-related risk associated with a single transport unit. The second analytical layer corresponds to the interaction level, which captures safety risks related to interactions between robots, human operators, and surrounding infrastructure. This subsystem uses indicators such as the Collision Risk Index, Emergency Stop Frequency and the Human–Robot Interaction Indicator to evaluate potentially hazardous operational situations.

The resulting output is the interaction risk indicator $R_{i}$, reflecting safety conditions within the logistics environment. The third analytical level represents the fleet/system perspective and focuses on coordination efficiency and operational stability of the entire robot fleet. The subsystem uses indicators such as the Congestion Index and System Coordination Reliability to evaluate system-wide operational risks related to traffic management and task coordination. Its output is the system-level risk indicator $R_{s}$, describing the overall operational stability of the autonomous transport system. All three analytical modules are implemented using the Mamdani fuzzy inference structure, widely applied in decision-support systems operating under uncertainty [79]. The inference process consists of fuzzification, rule-based inference, aggregation and defuzzification, enabling quantitative monitoring data from the Digital Twin environment to be transformed into interpretable linguistic risk assessments. During fuzzification, crisp input values obtained from the monitoring system are converted into fuzzy representations using predefined membership functions. Let x denote a crisp value of an input indicator acquired from the Digital Twin monitoring environment. The fuzzification process maps this value onto a fuzzy set using a membership function defined as

(6) $$\mu_{A}\left(x\right):X\;\to [0,\;1]$$

where *X* represents the universe of discourse of the input variable and $\mu_{A}\left(x\right)$ denotes the degree of membership of value *x* in the fuzzy set *A*. This transformation enables the model to represent gradual transitions between different system states rather than relying on rigid threshold values. In the proposed model, triangular and trapezoidal membership functions are used to represent linguistic terms describing operational conditions such as low, medium and high. The detailed interpretation of these linguistic variables for each indicator is presented in Table 8, Table 9 and Table 10.

Following the fuzzification stage, the fuzzy inference mechanism evaluates a set of expert-defined IF–THEN rules describing relationships between operational indicators and the corresponding risk level. These rules represent engineering knowledge regarding typical cause-and-effect relationships observed in autonomous mobile robot systems. Rather than being derived from statistical learning, the rules formalize operational expertise in a transparent and interpretable manner, allowing the reasoning process to remain fully explainable. A general form of a fuzzy rule can be expressed as

$$IF\;x_{1}\;is\;A_{1}\;AND\;x_{2}\;is\;A_{2}\ldots \;AND\;x_{n}\;is\;A_{n}\;\;THEN\;R\;is\;B$$

where *x*_1_, *x*_2_, …., *x*_n_ denote input indicators, *A*_1_, *A*_2_, …., *A*_n_ represent their linguistic states, and *R* corresponds to the resulting risk level represented by the fuzzy set *B*. The activation strength of each rule is calculated using the minimum operator applied to the membership values of the rule premises:

(7) $$\alpha_{k}=min(\mu_{A_{1}}\left(x_{1}\right),{\;\mu }_{A_{2}}\left(x_{2}\right),\;\ldots ,\;\mu_{A_{n}}\left(x_{n}\right))$$

where $\alpha_{k}$ denotes the activation degree of rule *k*. This approach allows the model to capture complex relationships among system indicators and to incorporate expert knowledge regarding operational risks in autonomous logistics systems.

The relative importance of the input indicators is incorporated into the fuzzy inference mechanism using weights obtained from the fuzzy Analytic Hierarchy Process described in Section 4.1. Let *w*_*i*_ denote the normalized weight assigned to indicator *i*. These weights are used to adjust the influence of individual indicators on the resulting risk evaluation by scaling their membership values according to the expression:

(8) $$\mu_{i}^{*}\left(x_{i}\right)=w_{i}\cdot \mu_{i}\left(x_{i}\right)$$

This weighting mechanism ensures that indicators identified as more critical from the expert perspective exert a stronger influence on the inference process and on the resulting risk estimation.

After the rule evaluation stage, the outputs of all activated rules are aggregated in order to obtain a combined fuzzy representation of the risk level for each analytical subsystem. The intermediate risk indicators obtained from the robot-level, interaction-level, and system-level subsystems are then integrated to produce the overall system risk evaluation. The final risk indicator *R_total_* is calculated using a weighted aggregation function:

(9) $$R_{total}=w_{r}R_{r}+w_{i}R_{i}+w_{s}R_{s}$$

where $w_{r},\;w_{i},$ and $w_{s}$ denote the weights assigned to the three analytical levels of the framework. These weights may also be determined using the fuzzy AHP procedure in order to reflect the relative importance of different risk dimensions.

The final stage of the inference process is defuzzification, which converts the aggregated fuzzy risk representation into a crisp numerical value that can be used for decision-making and system monitoring. In this study, defuzzification is performed using the centroid, or center-of-gravity, method, which is one of the most widely applied techniques in Mamdani fuzzy inference systems. The defuzzified risk value is calculated as:

(10) $$R=\frac{\int x\mu_{R}\left(x\right)dx}{\int \mu_{R}\left(x\right)dx}$$

where $\mu_{R}\left(x\right)$ represents the aggregated membership function of the output variable. The resulting value represents the final quantitative estimation of the risk level in the autonomous mobile robot-based internal logistics system. It can be used to support operational decision-making and proactive risk management strategies.

### 4.3. Risk Level Evaluation

The proper definition of linguistic variables, membership functions, and inference rules constitutes the knowledge base of the proposed fuzzy risk assessment model. These elements are developed to represent typical operational relationships observed in autonomous mobile robot systems and to translate engineering expertise into an interpretable computational model. Importantly, expert knowledge is used to define the model structure rather than to evaluate individual operational situations. Once the knowledge base has been established, the risk assessment process is driven automatically by operational KPI values obtained from the Digital Twin environment. In the proposed model, all input indicators are represented as linguistic variables describing the operational condition of the internal logistics system. This approach supports the representation of uncertainty and gradual transitions between system states, which is particularly important in dynamic autonomous transportation environments. For each input indicator, three linguistic states are defined: low, medium and high. Similarly, the output variable representing the overall system risk level is described using the states low risk, moderate risk and high risk. Their interpretation is based on expert knowledge, operational experience and typical performance thresholds observed in autonomous logistics systems. Because the indicators represent different operational phenomena, the interpretation of the low–medium–high scale is defined separately for each analytical level of the model. Table 8, Table 9, Table 10 and Table 11 present the semantic interpretation of linguistic variables used for robot-, interaction- and system-level indicators. The robot-level indicators describe the technical condition and operational reliability of individual autonomous mobile robots. The linguistic scale reflects the quality of robot performance and its ability to execute transport tasks without failures or disruptions.

Robot-level indicators primarily reflect the technical reliability and operational performance of the autonomous transport unit. Interaction-level indicators describe safety risks related to interactions between robots, humans and surrounding infrastructure within the internal logistics environment. These indicators reflect the dynamic nature of autonomous transportation systems, where safety depends not only on robot reliability but also on the quality of operational interactions.

These indicators reflect operational safety conditions and the level of potential hazards within the robot operating environment. System-level indicators represent the coordination efficiency and operational stability of the entire mobile robot fleet within the internal logistics system. They describe the overall performance of the transport system in terms of traffic management, coordination and logistics process stability.

System-level indicators, therefore, capture overall operational performance, coordination efficiency and transport flow stability within the internal logistics system. The final output of the fuzzy inference system is the overall risk level describing the safety and reliability condition of the autonomous internal transportation system.

The linguistic interpretation of risk levels enables decision-makers to quickly identify the severity of operational threats and implement appropriate mitigation strategies. The linguistic variables are represented using fuzzy membership functions defined within the normalized range [0, 1]. In the proposed model, triangular and trapezoidal membership functions are applied due to their simplicity and widespread use in fuzzy decision-support systems. The fuzzy rule base represents expert knowledge describing relationships between system indicators and the resulting risk level. The rules are defined separately for each subsystem of the hierarchical fuzzy model. Exemplary fuzzy rules for the robot-level subsystem are presented in Table 12.

The rules reflect typical operational relationships observed in autonomous logistics systems. For example, low condition monitoring indicators combined with poor navigation reliability increase the likelihood of operational failures and lead to a high-risk classification. Similar rule bases are defined for the interaction and system-level subsystems, considering factors such as collision probability and human–robot interactions, as well as transport route congestion. The fuzzy inference mechanism aggregates input variables according to the defined rule base and generates a fuzzy risk evaluation, which is subsequently transformed into a crisp value using the centroid defuzzification method. The resulting numerical risk index represents the overall safety and reliability condition of the autonomous internal transportation system and enables quantitative comparison of different operational states. From an operational perspective, the obtained risk level provides decision-makers with actionable information supporting maintenance planning, traffic management and safety supervision. Low risk values indicate stable and reliable operation, moderate risk levels signal emerging operational disturbances requiring monitoring or preventive adjustments, whereas high risk levels indicate critical conditions requiring immediate managerial intervention. Thus, the proposed fuzzy model serves as a decision-support tool integrating Digital Twin monitoring data with expert knowledge to provide interpretable and operationally meaningful risk assessments. The proposed knowledge base should, therefore, be interpreted as semi-static expert knowledge that defines the reasoning mechanism of the model. In practical implementations, the membership functions and rule base may be updated as additional operational experience becomes available or when the system configuration changes. This enables the framework to evolve over time while preserving the interpretability of the fuzzy inference process.

An important design principle adopted in the proposed rule base is the non-compensatory treatment of safety-critical indicators. In particular, the Collision Risk Index (CRI) is considered a dominant safety variable. Consequently, situations characterized by high collision risk cannot be fully compensated by favorable values of other operational indicators such as the emergency stop frequency or human–robot interaction. This modeling assumption reflects established safety engineering principles, according to which the presence of an imminent collision hazard should dominate the resulting risk assessment regardless of otherwise satisfactory system performance. Only in isolated situations, where CRI is high, but all remaining indicators remain consistently low, may the interaction risk be classified as moderate, reflecting increased vigilance rather than immediate critical conditions.

To demonstrate the practical applicability of the framework, the following section presents a case study illustrating the implementation of the proposed fuzzy risk assessment model in an autonomous internal logistics system with a fleet of mobile robots. All notation used in the model is consistent across robot-, interaction-, and system-level subsystems, where KPI inputs are denoted as $x_{i}^{norm}$, fuzzy membership values as *μ*(⋅), and subsystem outputs as *R_r_*, *R_i_*, and *R_s_*, aggregated into *R_total_*.

## 5. Case Study: Internal Transportation System with Mobile Robots

### 5.1. The Description of the Analyzed Logistics System and Identification of Operational Risk Scenarios

The analyzed system is an internal transport system operating within a manufacturing facility consisting of a high-bay warehouse and a production hall. The system relies on sensor-based monitoring and real-time operational data acquisition to support autonomous transportation processes between the warehouse and production areas. The system is served by a fleet of five mobile robots equipped with sensing systems for localization, navigation, obstacle detection and operational monitoring. Two robots operate between the warehouse and production hall, sharing space with forklifts and pedestrians, while three robots operate exclusively within the production hall along partially overlapping predefined routes. Safe operation, therefore, depends on reliable sensing and real-time environmental monitoring. Robot navigation is based on fixed maps supported by localization sensors and supervisory monitoring systems providing online visualization of robot states and positions. However, the system does not monitor forklifts or other moving obstacles, limiting its ability to account for all dynamic disturbances during traffic coordination.

Several recurring operational disturbances were identified in the analyzed system. In the warehouse area, localization instability and short-term Wi-Fi communication losses may reduce navigation accuracy and monitoring continuity. In the production hall, partially shared routes, temporary obstacles and avoidance maneuvers may lead to robot conflicts or deadlocks requiring human intervention. Consequently, the analyzed environment represents a shared human–robot intralogistics system in which safety and reliability depend on both robot performance and fleet coordination supported by continuous sensing and operational monitoring.

Based on the characteristics of the analyzed internal logistics system described above, a set of representative operational risk scenarios was identified. The analysis considered observed disturbances and system configuration, as well as interactions among robots, human operators, and manual transport equipment, with particular emphasis on factors affecting safety, coordination, and operational continuity. The analyzed system represents a shared human–robot intralogistics environment with partially overlapping routes, limited environmental sensing and fixed navigation maps. Consequently, operational risks arise not only from robot failures, but also from interaction dynamics, communication limitations and variability in material flow demand. The identified risk scenarios were grouped into five main categories: (i) localization and navigation disturbances, (ii) communication-related disruptions, (iii) traffic and interaction conflicts, (iv) operational flow variability, and (v) coordination and control limitations.

One of the key risk sources in the analyzed system is related to robot localization instability. In the warehouse area, particularly in zones with dense infrastructure, such as floor-level storage locations, temporary discrepancies between the actual and estimated robot positions may occur. These discrepancies result from limitations of the localization system and environmental conditions affecting sensor performance. Such deviations may lead to incorrect positioning during transport operations, increased stopping frequency, or conservative robot behavior, such as unnecessary slowdowns. In extreme cases, localization errors may contribute to navigation conflicts or trigger safety stops, reducing system throughput. This type of disturbance is particularly critical for robots operating between the warehouse and the production hall, where accurate positioning is required for safe interaction with other traffic participants.

Another important source of operational risk is associated with communication-related disruptions. The system relies on wireless communication to provide real-time monitoring and supervisory control; however, periodic short-term Wi-Fi signal losses have been observed. While robots are capable of continuing task execution locally, such communication interruptions reduce system transparency and limit the ability of operators to monitor system status in real time. As a result, delays in detecting abnormal situations or coordinating transport tasks may occur, particularly under conditions of increased operational load.

A significant group of risk scenarios emerges from interactions between robots, manually operated forklifts, and pedestrians. This is particularly relevant in the transport corridor connecting the warehouse and the production hall, as well as at designated crossing points where different traffic types intersect. In these areas, robots are required to reduce speed and yield to pedestrians, which introduces variability in travel times and may lead to temporary congestion. Within the production hall, where multiple robots operate on partially shared routes, additional conflict situations may arise during path crossing or obstacle avoidance maneuvers. Despite relatively wide transport paths, previous system observations indicate the occurrence of deadlock situations, in which robots block each other and require human intervention to resume operation. These situations highlight the limitations of local decision rules and the lack of coordinated traffic management mechanisms.

Operational risk is further influenced by variability in material flow and task generation. In the analyzed system, transport tasks are not strictly periodic but depend on the dynamics of production processes. This is particularly evident in the handling of finished products and production waste, where transport demand is triggered by process conditions rather than fixed schedules. Such variability may lead to temporary mismatches between transport demand and system capacity. For instance, delays in removing finished products from production stations may result in interruptions of production processes due to limited buffer capacity, whereas periods of low demand may lead to underutilization of transport resources. Although the introduction of mobile robots aims to improve responsiveness and reduce dependence on manual transport, the lack of adaptive task allocation mechanisms limits the system’s ability to fully respond to dynamic demand patterns.

Finally, the identified risk scenarios are strongly influenced by limitations of the supervisory and control system. While the system provides real-time visualization of robot states and estimated positions, it does not account for all dynamic elements present in the environment, such as the positions of forklifts or temporary obstacles. This limited situational awareness reduces the ability to anticipate conflicts and results in predominantly reactive control strategies. Additionally, the use of fixed navigation maps without dynamic route replanning reduces system flexibility in responding to disturbances, such as blocked paths or local congestion. These constraints contribute to the occurrence of previously observed issues, including inefficient routing, delays and deadlock situations.

Overall, the identified operational risk scenarios reflect the complexity of interactions within the analyzed logistics system and highlight the presence of both technical and organizational sources of disturbances. Importantly, many of these risks are characterized by uncertainty, variability, and context-dependent behavior, which makes them difficult to assess using deterministic approaches. Therefore, in the next section, these scenarios are translated into a structured set of input variables for the proposed fuzzy logic-based risk evaluation model, enabling the integration of quantitative system data with qualitative expert knowledge in the assessment of operational risk.

### 5.2. Case Study Data Acquisition and Implementation

The analyzed case study is based on representative operational data obtained within a collaborative research project conducted together with the manufacturer of autonomous mobile robots intended for internal logistics applications. The objective of the project was the development and validation of an autonomous mobile robot capable of operating in warehouse environments. The experimental campaign involved two autonomous mobile robots operating in a controlled warehouse-like environment. The collected data consisted of two complementary datasets: (i) a short-term experimental observation dataset used to analyze representative operational scenarios and validate Digital Twin-based monitoring, and (ii) a cumulative operational dataset obtained from the complete validation campaign and used for reliability- and safety-related KPI calculation. This distinction was introduced because indicators such as the Mean Time Between Failures (MTBF) and Emergency Stop Frequency (ESF) require a sufficiently long exposure period to provide meaningful estimates.

The experimental observation phase covered five consecutive working days, during which the robots operated for approximately 4–5 h per day. This resulted in approximately 22 h of direct experimental observation per robot. During this period, the robots executed several hundred transport tasks under varying operational conditions, including navigation maneuvers, task allocation scenarios, human–robot interaction situations, and safety-related events.

In addition to the experimental observation period, cumulative operational logs collected throughout the complete validation campaign were used for reliability-oriented calculations. The cumulative dataset included approximately 740 robot operating hours and four recorded hardware failures, allowing the calculation of MTBF, according to Equation (12). Similarly, safety-related event indicators, including the Emergency Stop Frequency, were calculated using the accumulated operational exposure time available from the validation logs. The main characteristics of the datasets used for KPI calculation are summarized in Table 13.

The collected dataset represents an experimentally validated operational scenario complemented by cumulative validation records. The objective of the dataset was not to statistically characterize the long-term reliability of a commercial AMR fleet, but to demonstrate the applicability of the proposed Digital Twin-based fuzzy risk assessment methodology. Therefore, short-term operational observations were used to capture representative system states, while cumulative operational records were used only for indicators requiring longer-term exposure, such as the MTBF and event-frequency measures.

This approach follows common practice in reliability engineering, where instantaneous operational states and cumulative reliability indicators are derived from datasets with different temporal resolutions. Consequently, the proposed framework preserves both operational realism and methodological consistency by combining real-time monitoring information with reliability-oriented historical records.

These data were acquired from the robot monitoring system and onboard sensing infrastructure, including LiDAR sensors, vision cameras, localization modules and communication logs. In addition, during the project, the operational environment of the robots was modeled using the Gazebo simulation environment, while warehouse material flow and transport processes were analyzed using FlexSim. The individual data sources served different purposes within the proposed Digital Twin architecture. Physical robot telemetry provided direct measurements of robot condition and safety-related events, whereas the Fleet Management System supplied operational records related to task execution and fleet coordination. The Gazebo environment was used primarily for validating navigation behavior and collision-related scenarios, while FlexSim supported the analysis of warehouse-level traffic conditions, congestion phenomena, fleet utilization, and simulated disturbance scenarios. The relationship between data sources and collected information is summarized in Table 14.

The separation of data sources ensures that the proposed framework does not treat simulation outputs as direct replacements for physical measurements. Instead, simulation environments complement experimental data by providing additional information regarding system-level behavior and allowing controlled analysis of operational scenarios that are difficult to reproduce during physical experiments.

Since the primary objective of this paper is to validate the proposed decision-support methodology rather than to statistically evaluate the long-term performance of a specific warehouse installation, the KPI values presented in Section 5.5 were calculated for a defined observation window selected from the experimental dataset. The selected observation window corresponded to a four-hour operational period representing a stable warehouse operating cycle with multiple transport tasks, navigation events, safety interactions, and fleet coordination activities.

Although the Digital Twin architecture enables continuous acquisition of operational data, the proposed fuzzy assessment framework evaluates system status based on aggregated KPI values calculated over a predefined observation horizon. This approach reflects practical engineering applications, where operational decisions are commonly supported by periodically updated performance indicators rather than by individual instantaneous measurements. This approach ensures that the presented case study reflects realistic operational conditions while maintaining clarity and reproducibility of the proposed methodology.

The Digital Twin architecture implemented in this study follows a three-layer structure designed to integrate heterogeneous data sources and enable consistent KPI computation for risk assessment. The first layer corresponds to the data acquisition layer, which includes onboard robot sensing systems (LiDAR, vision cameras, and localization modules), robot internal diagnostics, and logs from the fleet management system, capturing task execution, navigation events, and safety-related incidents. The second layer represents the data integration and synchronization layer, which aggregates and aligns heterogeneous data streams originating from both the physical system and simulation environments. In this study, simulation-based data from the Gazebo environment and logistics flow models developed in FlexSim are synchronized with real operational logs through time-stamped event alignment and unified data structuring within a common data schema. The third layer corresponds to the analytics and decision-support layer, where processed data are transformed into key performance indicators (KPIs) and subsequently used as inputs to the fuzzy risk assessment model. The contribution of each data source to the individual KPIs is summarized in Table 15. The robot-level indicators are primarily derived from physical telemetry and operational logs and the interaction-level indicators combine robot sensing information with navigation and safety event records, while the system-level indicators additionally incorporate fleet management and warehouse simulation outputs.

This data-source mapping provides traceability between the Digital Twin layer and the subsequent fuzzy evaluation process. Consequently, each linguistic assessment obtained from the fuzzy inference system can be related to a clearly defined set of operational measurements and simulation-supported analyses.

Data processing follows a hybrid synchronization strategy. Real-world operational data are recorded in an event-driven manner with sub-second resolution at the sensor and system log level, while simulation outputs from Gazebo and FlexSim are generated according to discrete simulation time steps. These heterogeneous data streams are aggregated over a unified temporal window to ensure comparability at the KPI level. Consequently, the Digital Twin operates as a semi-real-time system, enabling continuous data acquisition and monitoring at the source level, while the decision-support analysis is performed on aggregated KPI representations over a defined observation horizon. This hybrid configuration allows the framework to combine experimentally collected operational data with simulation-based system representations, ensuring both realism and controllability of the analyzed case study while maintaining methodological consistency across data sources.

In addition to operational data, the proposed framework incorporates structured expert knowledge during the model development stage. Expert knowledge was elicited through a series of technical consultations conducted within the research project involving both engineers from the mobile robot manufacturer and academic researchers specializing in autonomous transportation systems, logistics, reliability engineering, and fuzzy decision-support methods. The expert panel consisted of 3 specialists, including 1 industrial engineer directly involved in the development and testing of the AMR platform and 2 academic researchers with expertise in autonomous logistics systems and risk assessment. The separation between data-driven evaluation and expert-based model configuration is maintained throughout the framework. Operational data determine the current risk state of the AMR system, whereas expert knowledge is used only to define indicator importance, linguistic interpretation, and fuzzy inference rules.

The experts did not evaluate individual operational scenarios. Instead, their role was limited to defining the structural knowledge embedded in the framework. Specifically, expert judgments were used to:

- Establish pairwise comparisons between KPIs within the fuzzy AHP procedure,
- Define linguistic interpretations of the monitored indicators,
- Formulate the fuzzy IF–THEN rule base describing typical operational relationships observed in AMR-based logistics systems.

Once the knowledge base was established, all subsequent risk evaluations were performed automatically using KPI values calculated from Digital Twin operational data. Consequently, expert knowledge influences the framework indirectly through the model structure, whereas the final risk assessment depends primarily on the monitored operational state of the analyzed system.

To reduce individual subjectivity, expert judgments were collected independently and subsequently aggregated before constructing the final pairwise comparison matrices. The consistency of the resulting judgments was later evaluated using the consistency ratio (CR), while additional robustness analyses based on perturbation scenarios were performed in Section 5.4 to assess the sensitivity of the framework to plausible variations in expert opinions.

### 5.3. The Application of the Proposed Framework

The proposed framework was applied to a real-world-inspired case study of an internal logistics system based on a fleet of autonomous mobile robots operating in a warehouse environment. The system performs repetitive transport operations among storage zones, picking stations and dispatch areas. The fleet consists of multiple AMRs managed by a centralized fleet management system, operating in a shared space with human workers and other infrastructure elements. The environment is characterized by dynamic task allocation, variable traffic intensity and the presence of human–robot interaction zones, which makes it suitable for evaluating risk under realistic operational conditions.

The implementation of the framework required the integration of the physical system with a Digital Twin environment. In the analyzed case, the Digital Twin was implemented as a data-integrating and monitoring layer connected to three main data sources: (i) on-board robot systems, (ii) fleet management system, and (iii) warehouse management system. The Digital Twin continuously collected and synchronized operational data at the source level, whereas the risk evaluation presented in this study was performed using KPI values aggregated over predefined observation windows. These data streams were processed to generate structured information used for KPI calculation.

Consistent with the conceptual framework presented in Section 3, the same set of KPI was adopted in the case study, covering three analytical levels: robot level, interaction level and system level. At this stage, the indicators were operationalized using data available in the Digital Twin environment and interpreted in a way that is consistent with the linguistic variables defined in Section 4.3.

To ensure full reproducibility of the KPI calculation procedure, each indicator was explicitly defined in terms of raw input variables, mathematical formulation, and calculation parameters. In particular, composite indicators requiring aggregation of multiple information sources were described using predefined weighting coefficients, whereas ratio-based indicators were calculated directly from operational measurements without additional weighting factors. Table 16 summarizes the complete KPI formulation adopted in the case study, including raw input variables, weighting coefficients where applicable, and numerical values used for KPI calculation.

The formulation presented in Table 16 establishes a direct link between the Digital Twin data layer and the subsequent fuzzy risk assessment procedure. Composite indicators, including CMI, CRI, and CI, integrate several complementary measurements through predefined weighting coefficients. The adopted coefficients were selected based on engineering interpretation of the relative contribution of individual variables to the monitored phenomenon. For example, in the Collision Risk Index, near-collision frequency was assigned a higher contribution than minimum separation distance because the occurrence of near-miss events directly represents hazardous operational situations, while distance measurements provide additional information regarding the available safety margin. Similarly, the Congestion Index assigns greater importance to waiting time because it directly reflects operational degradation perceived by the logistics system.

The weighting coefficients used in the composite indicators were not obtained from the fuzzy AHP procedure, as their purpose differs from the subsequent indicator prioritization. The coefficients α, β and γ define the internal structure of individual composite KPIs, i.e., the relative contribution of input variables within a single indicator, whereas fuzzy AHP weights determine the relative importance of different KPIs within the overall risk assessment model. Therefore, the coefficients were predefined based on engineering knowledge of the monitored phenomena and characteristics of the AMR operational process. For the Condition Monitoring Index, higher importance was assigned to battery and motor-related variables because they directly reflect the technical state of the robot. For the Collision Risk Index, near-collision frequency was given higher importance than minimum separation distance due to its direct relation with safety-critical events. Similarly, the Congestion Index assigned a higher contribution to waiting time because it directly represents operational degradation perceived at the system level.

In contrast, indicators such as the MTBF, HRI, ESF and SCR were calculated using direct ratio-based formulations and, therefore, do not require additional weighting coefficients. Their values directly represent reliability, interaction intensity, safety event frequency, or coordination performance based on measured operational quantities.

At the robot level, the Condition Monitoring Index (CMI) was calculated as a composite measure reflecting the technical condition of the robot, based on diagnostic signals, such as battery status, motor load and sensor performance:

(11) $$CMI=\;\sum_{k=1}^{p}\alpha_{k}\cdot x_{k}$$

where $x_{k}$ represents normalized diagnostic variables and $\alpha_{k}$ are weighting coefficients reflecting their importance.

From the perspective of linguistic interpretation, this indicator reflects the degree of technical degradation of the robot. Lower values correspond to deteriorating condition (low), while higher values indicate stable and healthy operation (high).

The Mean Time Between Failures (MTBF) was determined using operational and maintenance records:

(12) $$MTBF=\;\frac{T_{oper}}{N_{f}}$$

where $T_{oper}$ denotes cumulative operating time and $N_{f}$ is the number of recorded failures within the analyzed period.

This indicator reflects operational reliability over time. Lower MTBF values correspond to frequent failures (low), whereas higher values indicate stable long-term operation (high).

Navigation Reliability was determined as the ratio of successfully executed trajectories ($N_{success})$ to total planned trajectories ($N_{total})$, taking into account deviations and replanning events:

(13) $$NR=\;\frac{N_{success}}{N_{total}}$$

This KPI reflects the ability of the robot to follow planned trajectories without deviations. Lower values correspond to unstable navigation and frequent corrections (low), while higher values indicate consistent and accurate navigation (high).

The Task Completion Rate was calculated as:

(14) $$TCR=\;\frac{N_{completed}}{N_{assigned}}$$

where $N_{completed}$ represents successfully completed transport tasks and $N_{assigned}$ denotes all assigned tasks.

This indicator reflects operational efficiency. Lower values indicate disruptions in task execution (low), whereas higher values represent stable and efficient task completion (high).

At the interaction level, three indicators were used. The Collision Risk Index (CRI) was defined as a composite measure based on proximity events, near-misses and minimum separation distances:

(15) $$CRI=\;\beta_{1}\cdot f_{near}+\beta_{2}\cdot d_{min}^{-1}$$

where $f_{near}$ represents the frequency of near-collision events and *d_min_* is the average minimum distance to obstacles or humans.

This indicator reflects the level of exposure to collision-related hazards. Higher values correspond to more frequent proximity events and reduced safety margins (high risk), whereas lower values indicate safe operation (low risk).

The Emergency Stop Frequency (ESF) was calculated as the number of emergency stop events ($N_{stop})$ per unit of operating time ($T_{oper})$:

(16) $$ESF=\;\frac{N_{stop}}{T_{oper}}$$

This KPI reflects the frequency of safety-critical interruptions. Higher values indicate unstable or hazardous operating conditions (high), while low values correspond to stable operation (low).

The Human–Robot Interaction Indicator (HRI) was determined based on the proportion of time robots operate in zones with human presence:

(17) $$HRI=\;\frac{T_{human}}{T_{oper}}$$

This indicator reflects the intensity of interactions between robots and human operators. Higher values indicate frequent interactions and increased operational complexity (high), while lower values correspond to limited interaction (low).

At the system level, two indicators were used. The Congestion Index (CI) was calculated based on average waiting times ($t_{wait})$ and queue lengths ($q_{length}$) observed in shared transport zones:

(18) $$CI=\;\gamma_{1}\cdot t_{wait}+\gamma_{2}\cdot q_{length}$$

This KPI reflects traffic conditions within the system. Higher values indicate congestion, delays, and blocked routes (high), whereas lower values correspond to smooth traffic flow (low).

System Coordination Reliability (SCR) was defined as the ratio of successfully executed coordinated tasks ($N_{coord\_success})$ to all coordination attempts ($N_{coord\_total})$, as follows:

(19) $$SCR=\;\frac{N_{coord\_success}}{N_{coord\_total}}$$

This indicator reflects the effectiveness of fleet coordination. Lower values indicate coordination problems (low), while higher values correspond to efficient system-level operation (high).

To improve reproducibility, all input variables used for KPI calculation and corresponding numerical parameters are summarized in Table 16. Composite indicators (CMI, CRI, and CI) require predefined weighting coefficients, which were explicitly reported. Ratio-based indicators (MTBF, NR, TCR, ESF, HRI, and SCR) were calculated directly from measured operational quantities and, therefore, do not include additional weighting parameters. All KPI values were later normalized to the range [0, 1] in order to ensure comparability (see Section 5.5). Table 17 presents the calculated values together with their qualitative interpretation based on the linguistic scales defined in Section 4.3.

The Digital Twin environment enables continuous data acquisition, resulting in time-dependent representations of all monitored variables. Therefore, the KPI values used in the analysis were not derived from single time instances but were calculated over a defined observation window. In this study, a representative operational period was selected, and all indicators were aggregated over this time horizon in order to capture the typical system behavior under realistic conditions. Although the Digital Twin continuously acquires and synchronizes operational data in real time, the objective of the present case study was not to evaluate the temporal dynamics of risk evolution but to validate the proposed decision-support methodology under representative operating conditions. Therefore, the KPI values were aggregated over a predefined observation window before being used as inputs to the fuzzy AHP and fuzzy inference models. In an operational deployment, the same KPI calculation procedure could be performed repeatedly using sliding or rolling time windows, allowing the risk level to be continuously updated as new monitoring data becomes available. Consequently, the aggregation applied in this study represents a validation strategy rather than a limitation of the proposed framework itself.

Depending on the nature of each KPI, different aggregation methods were applied. For condition- and reliability-related indicators, such as the Condition Monitoring Index and MTBF, time-averaged or cumulative measures were used. Performance-related indicators, including the Navigation Reliability and the Task Completion Rate, were calculated as ratios over the entire observation period. For interaction-related indicators, such as the Collision Risk Index and Emergency Stop Frequency, frequency-based aggregation was applied. System-level indicators, including congestion and coordination reliability, were derived using averaged or cumulative measures reflecting traffic dynamics over time.

This approach ensures that the calculated KPI values represent stable and meaningful descriptors of system performance, while preserving the dynamic nature of the Digital Twin data. The aggregated values were subsequently used for linguistic interpretation and as inputs to the fuzzy AHP and fuzzy inference models.

The calculated KPI values provide a quantitative description of the system’s operational state, while their linguistic interpretation enables consistent mapping to the fuzzy variables defined in Section 4.3. This step constitutes a critical link between the data-driven Digital Twin environment and the knowledge-based fuzzy inference model.

The resulting set of weighted and linguistically interpreted indicators forms the input to the subsequent stages of the analysis, including the determination of indicator importance using the fuzzy AHP method (Section 5.4) and the evaluation of the overall risk level using the fuzzy inference system (Section 5.5). Following this, to ensure consistency across all indicators used in the fuzzy inference model, a unified interpretation rule was adopted. All KPI variables were defined such that higher normalized values consistently correspond to improved system performance and/or reduced operational risk, depending on the indicator type. Specifically, benefit-type indicators (e.g., CMI, MTBF, NR, TCR, and SCR) are positively oriented, whereas cost-type indicators (e.g., CRI, ESF, HRI, and CI) are inverted during normalization to ensure that higher normalized values uniformly represent more favorable system states. This guarantees consistent semantic interpretation of all inputs within the fuzzy inference system and prevents ambiguity in rule activation and aggregation processes. Furthermore, all subsystem outputs (*R_r_*, *R_i_*, and *R_s_*) are expressed on a common [0, 1] risk scale, ensuring their comparability and enabling coherent computation of the overall risk index *R_total_*.

### 5.4. The Results of the Fuzzy AHP Analysis

The fuzzy AHP procedure described in Section 4.1 was applied to determine the relative importance of the key performance indicators. The analysis was performed hierarchically, including (i) comparison of analytical levels and (ii) comparison of indicators within each level. All pairwise comparisons were provided by the expert panel described in Section 5.2 and expressed using the linguistic scale defined in Table 7. The individual judgments were aggregated to obtain a single fuzzy comparison matrix for each hierarchy level before applying the fuzzy AHP procedure.

To ensure methodological rigor, the consistency of expert judgments was verified using the classical consistency ratio (CR), calculated based on the modal (defuzzified) values of the fuzzy numbers. In the following results, the reported values correspond to the consistency ratio (CR), which was directly used to assess the acceptability of expert judgments.

Due to space limitations and to maintain clarity of the main text, the full fuzzy pairwise comparison matrices and intermediate calculations are provided in Appendix A. This includes the detailed fuzzy comparison matrices, aggregation of expert judgments and intermediate synthetic extent values. The key results of the fuzzy AHP analysis are presented below.

The first stage of the analysis involved determining the relative importance of the three analytical levels: robot, interaction and system. The results indicate that the interaction level has the strongest influence on the overall risk, followed by the robot level, while the system-level indicators have a slightly lower but still significant contribution. Specifically, the derived weights were 0.307 for the robot level, 0.520 for the interaction level, and 0.173 for the system level. This distribution reflects the fact that, in autonomous logistics environments, risk is strongly influenced not only by technical reliability but also by dynamic interactions between robots, humans, and infrastructure. For this comparison matrix, the consistency ratio was equal to CR = 0.1467, exceeding the conventional AHP acceptance threshold of 0.1. This indicates a moderate level of inconsistency in the aggregated expert judgments. Therefore, this value was treated as a methodological limitation of the weighting process and was not interpreted as fully satisfying the classical AHP consistency requirement. However, because the analyzed hierarchy represents a complex safety assessment problem involving uncertain and qualitative expert evaluations, the obtained CR value was further examined through a dedicated sensitivity analysis rather than being interpreted independently. The complete summary of consistency verification results for all hierarchical levels, together with the corresponding robustness analysis, is provided in Appendix A (Table A14).

At the next stage, local weights were determined for indicators within each analytical group. For the robot-level subsystem, the results show that the Condition Monitoring Index (0.423) and the Mean Time Between Failures (0.315) dominate over the Navigation Reliability (0.151) and the Task Completion Rate (0.111), indicating that technical condition and reliability-related indicators are the most influential factors at this level. In the interaction-level subsystem, the Collision Risk Index (0.601) is identified as the most critical factor, followed by the Emergency Stop Frequency (0.276) and the Human–Robot Interaction Indicator (0.123), reflecting the dominant role of safety-related interaction effects. For the system-level subsystem, the Congestion Index (0.644) has a higher importance than the System Coordination Reliability (0.356), indicating that traffic-related effects are the primary driver of system-level risk. The consistency ratio for the robot-level matrix was CR = 0.104, while the interaction-level matrix resulted in CR = 0.116. These values exceed the conventional threshold of 0.1 and were, therefore, further examined through sensitivity analysis to verify their impact on the final priority structure. For the 2 × 2 system-level matrix, consistency is inherently satisfied (RI = 0), as expected. The final stage involved calculating the global weights of all indicators by combining local weights with the weights of their respective analytical levels. These global weights represent the final importance of each KPI in the overall risk assessment model. The global weights were calculated as the product of the corresponding local and level weights, i.e.:

(20) $$w_{global}=w_{level}\cdot w_{local}\;$$

The results are presented in Table 18.

The sum of all global weights equals 1.0, confirming the correctness of the normalization procedure. The obtained results indicate that the most influential indicators in the overall risk assessment are the Collision Risk Index, Emergency Stop Frequency and Condition Monitoring Index. These factors are directly associated with safety-critical events and the technical condition of the system, confirming their dominant role in shaping operational risk in AMR-based logistics systems. The resulting set of global weights provides a quantitative representation of the relative importance of the analyzed indicators and constitutes a key input to the fuzzy inference model developed in the next stage of the analysis. By integrating expert knowledge with data-driven monitoring, the weighting process ensures that the subsequent risk evaluation reflects both the structural characteristics of the system and the practical relevance of individual performance indicators. It should be emphasized that the consistency ratios exceeding the conventional threshold of 0.1 represent a limitation of the expert-based weighting process. Therefore, the robustness of the obtained priority structure was not assessed solely based on CR values but was additionally verified through systematic perturbation-based sensitivity analysis. The results presented below demonstrate that moderate variations in expert judgments do not alter the ranking structure of the indicators, confirming the stability of the derived weights.

To address the consistency limitation identified by the CR analysis and to verify whether the observed inconsistency affects the final prioritization results, a comprehensive sensitivity analysis was conducted across all hierarchical levels of the model. The analysis examines the stability of the derived priority structures under plausible perturbations of expert judgments at (i) the top-level analytical hierarchy (robot–interaction–system), (ii) the KPI subsystem level, and (iii) the interaction-level safety subsystem. Although some consistency ratios exceed the conventional threshold of 0.1, this does not automatically invalidate the obtained priorities, provided that the robustness of the resulting ranking is systematically verified. In expert-based multi-criteria decision problems, moderate deviations from the ideal consistency level may occur due to the qualitative nature of pairwise comparisons and the complexity of the evaluated system. Therefore, instead of accepting the obtained CR values without further verification, the robustness of the resulting priority structure was examined through a systematic sensitivity analysis.

To directly address the potential influence of the elevated CR values on the final decision structure, a systematic sensitivity analysis was performed. The objective was not to artificially reduce the inconsistency measure, but to verify whether plausible variations in expert judgments could change the resulting priority ranking. In the first step, a ±10% perturbation scenario was applied to the fuzzy pairwise comparison matrix of analytical levels. This modification reflects realistic variability in expert judgments while preserving the structure of the decision problem. The recomputed weights show only minor deviations from the baseline solution, with the interaction level remaining the most important factor, followed by the robot and system levels. The obtained weights were 0.327 (robot), 0.505 (interaction), and 0.168 (system), compared to the baseline values of 0.307, 0.520, and 0.173, respectively.

In the second scenario, a modified perturbation was introduced by adjusting selected pairwise comparison values within a limited range to further test model stability under alternative but still plausible expert assumptions. The resulting weights (robot = 0.309, interaction = 0.509, and system = 0.182) remain very close to the baseline solution, confirming structural stability. In this case, the consistency ratio decreased to CR = 0.1127 from the baseline value of CR = 0.1467, indicating improved coherence of expert judgments under the perturbed configuration, although still slightly above the conventional threshold of 0.1.

Across both scenarios, no rank reversals were observed, confirming the stability of the analytical hierarchy. These results demonstrate that the proposed weighting scheme is robust with respect to moderate variations in expert judgments and preserves a consistent prioritization structure under uncertainty.

To further validate the robustness of the model, sensitivity analysis was also performed at the KPI subsystem level. A perturbed fuzzy pairwise comparison matrix was constructed for the robot-level indicators, and the full fuzzy AHP procedure was reapplied. The obtained results confirm the stability of the ranking structure, with the Condition Monitoring Index (0.379) and the Mean Time Between Failures (0.334) remaining the most important indicators, followed by the Navigation Reliability (0.165) and the Task Completion Rate (0.123). The consistency ratio of the perturbed model (CR = 0.10933) remains close to the baseline level. Although it still exceeds the conventional threshold of 0.1, the unchanged ranking structure confirms that the observed inconsistency does not substantially affect the decision priorities.

Overall, the results confirm that the proposed fuzzy AHP model is robust not only at the system hierarchy level but also within individual KPI subsystems. No rank reversals or structural changes were observed, demonstrating the stability of the derived weighting scheme under reasonable perturbations of expert judgments.

Finally, a dedicated sensitivity analysis was conducted for the interaction-level subsystem, which represents the most safety-critical component of the proposed framework. The pairwise comparison matrix for the Collision Risk Index (CRI), Emergency Stop Frequency (ESF), and Human–Robot Interaction Indicator (HRI) was slightly modified by reducing the dominance intensity between CRI and ESF, reflecting plausible variability in expert assessments.

The recalculated weights confirm stable ranking of interaction-level indicators: CRI = 0.543, ESF = 0.333, and HRI = 0.124. No rank reversals were observed, and the overall priority structure remained unchanged compared to the baseline model. The consistency ratio improved to CR = 0.10486 (from CR = 0.116), indicating slightly higher coherence of expert judgments under the perturbed scenario.

Among all analyzed levels, the interaction subsystem exhibits the highest sensitivity to judgment perturbations, which is consistent with its role as the most safety-critical component of the system. Nevertheless, even in this case, the ranking stability is preserved, confirming the reliability of the proposed decision model.

Thus, although the classical consistency criterion was not fully achieved in all comparison matrices, the obtained weighting structure demonstrated stability under plausible uncertainty scenarios. The consistency of ranking results across all scenarios confirms that the derived prioritization can be reliably used as input to the subsequent fuzzy inference-based risk assessment model.

### 5.5. Risk Level Evaluation Using the Fuzzy Inference System

The final stage of the proposed framework consists of evaluating the overall operational risk using the hierarchical Mamdani fuzzy inference system described in Section 4.2. This stage integrates (i) normalized KPI values obtained from the Digital Twin environment and (ii) indicator weights derived from the fuzzy AHP analysis.

#### 5.5.1. Normalization and Fuzzification of KPI Values

The proposed fuzzy inference model requires all input variables to be expressed on a common scale. However, not all key performance indicators require an additional normalization step. The indicators inherently defined as ratios or composite indices bounded within the interval [0, 1] were directly used as fuzzy inputs, whereas indicators expressed in physical units were transformed to the normalized interval [0, 1] before fuzzification.

Consequently, CMI, NR, TCR and SCR were used directly because their mathematical formulations naturally produce dimensionless values within the required interval. Cost-oriented indicators expressed within the interval [0, 1] (CRI and HRI) required only orientation adjustment using Equation (22), whereas indicators expressed in physical units (ESF and CI) required both range normalization and orientation adjustment.

For benefit-type indicators (higher value = better performance), normalization was performed as:

(21) $$x_{i}^{norm}=\frac{x_{i}-x_{min}}{x_{max}-x_{min}}\;$$

For cost-type indicators (higher value = higher risk), the inverse transformation was applied:

(22) $$x_{i}^{norm}=\frac{{x_{max}-x}_{i}}{x_{max}-x_{min}}\;$$

The normalization procedure adopted for each KPI is summarized in Table 19. For indicators already expressed in the interval [0, 1], no range scaling was required. Cost-oriented indicators were additionally transformed using Equation (22) to ensure a consistent semantic interpretation, whereas indicators expressed in physical units required both range normalization and orientation adjustment.

As a result, the normalized KPI values used as inputs to the fuzzy inference system are given in Table 20.

The adopted normalization strategy distinguishes between inherently normalized indicators and variables expressed in physical units. This approach avoids unnecessary transformations of ratio-based indicators while ensuring comparability among all fuzzy inputs. The normalization limits reported in Table 19 provide full traceability of the scaling procedure and distinguish between limits derived from experimental observations, cumulative operational records, simulation-supported analyses, and manufacturer-validated operating constraints. Consequently, every KPI entering the fuzzy inference model is expressed on a consistent numerical scale while preserving its physical interpretation.

#### 5.5.2. Fuzzification of Normalized KPI Values

The fuzzification stage constitutes a critical step in the proposed risk assessment framework, as it enables the transformation of normalized quantitative KPI values into linguistic representations that can be processed within the fuzzy inference system. Following the normalization procedure described in Section 5.5.1, each KPI is represented as a crisp value *x*_*i*_ ∈ [0, 1]. These values are subsequently mapped onto fuzzy sets using predefined membership functions, in accordance with Equation (6).

**Figure 3:**
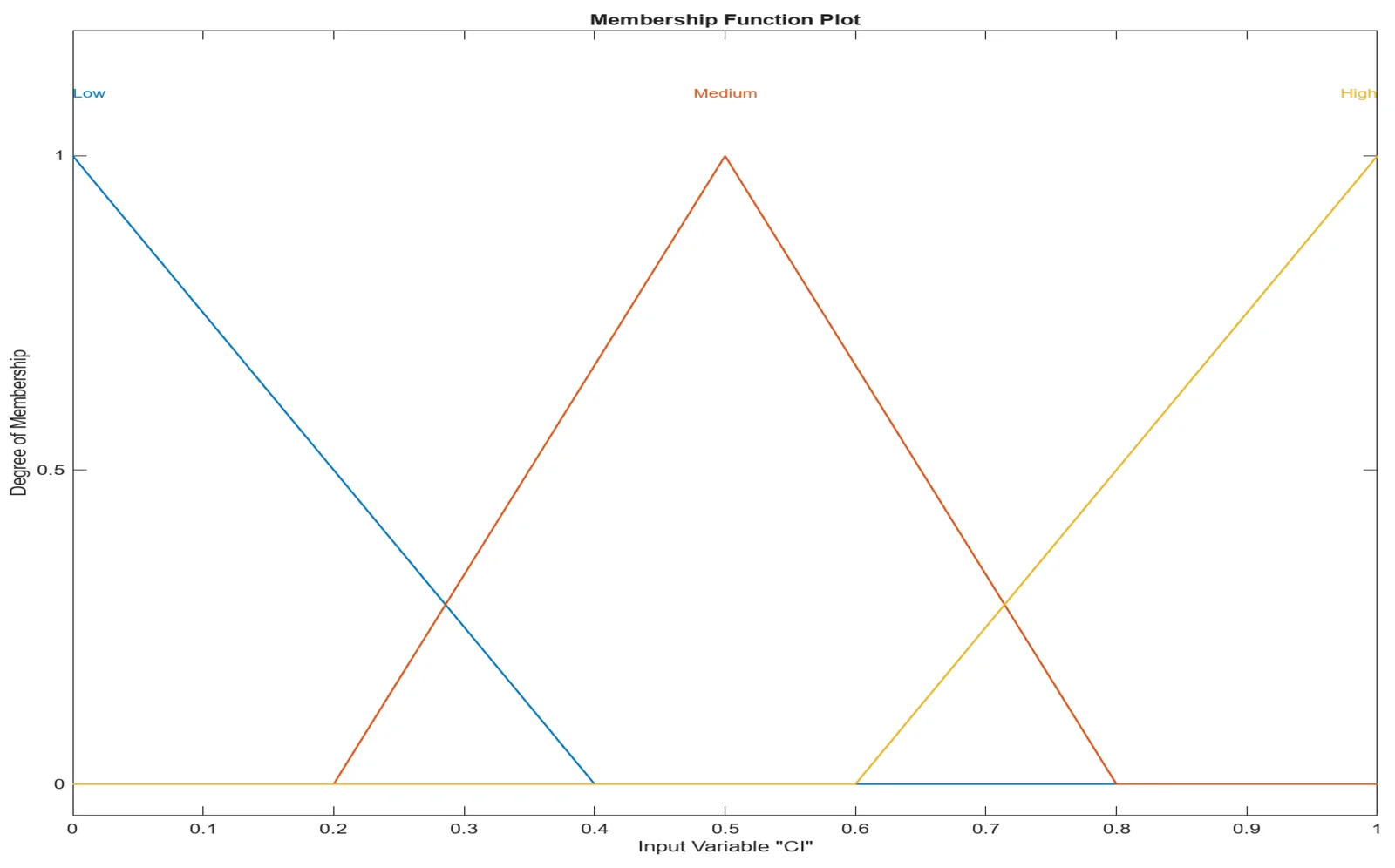
The membership functions adopted in the proposed case study.

In this study, all input variables are described using triangular membership functions, due to their simplicity and interpretability. The functions were defined consistently across indicators within the normalized domain [0, 1], while preserving the semantic interpretation introduced in Section 4.3. The adopted membership functions are presented in Figure 3.

This configuration ensures smooth transitions between adjacent linguistic states and allows partial membership in multiple sets, which reflects the uncertainty and gradual nature of system behavior in autonomous logistics environments. For each KPI, the normalized value *x*_*i*_ is evaluated against all three membership functions, resulting in a vector of membership degrees:

(23) $$[\mu_{Low}\left(x_{i}\right),\mu_{Medium}\left(x_{i}\right),\mu_{High}\left(x_{i}\right)]\;$$

Importantly, membership degrees in fuzzy logic represent the degree of compatibility between a numerical value and a linguistic concept and should not be interpreted as probabilities. Therefore, their values do not necessarily sum to one. A single KPI value may simultaneously belong to more than one fuzzy set, which enables the representation of intermediate operational states.

The membership degrees used in the subsequent inference stage were calculated directly from the implemented fuzzy inference systems using the predefined triangular membership functions. Therefore, the values presented in Table 21 represent the actual fuzzification outputs used for rule activation.

As the interaction-level subsystem was identified as the most critical component in the analyzed case study, its fuzzification process is presented in detail. For the Collision Risk Index (CRI), the normalized value is *x_CRI_* = 0.62. Evaluation against the membership functions results in:

- $\mu_{Low}\left(0.62\right)=$ 0;
- $\mu_{Medium}\left(0.62\right)$= 0.60;
- $\mu_{High}\left(0.62\right)=$ 0.05.

Similarly, the Emergency Stop Frequency (ESF) value *x_ESF_* = 0.91 is classified predominantly as high:

- $\mu_{Low}$(0.91) = 0;
- $\mu_{Medium}$(0.91) = 0;
- $\mu_{High}$(0.91) = 0.775.

while the Human–Robot Interaction Indicator (HRI) value *x_HRI_* = 0.59 corresponds to:

- $\mu_{Low}$(0.59) = 0;
- $\mu_{Medium}$(0.59) = 0.70;
- $\mu_{High}$(0.59) = 0.

These results demonstrate that the operational state is represented by overlapping fuzzy conditions rather than a single deterministic category. In particular, the interaction-level subsystem is characterized by elevated emergency-stop frequency combined with medium collision and human–robot interaction conditions, which directly influences the subsequent rule activation process.

The summary of fuzzification results is given in Table 21.

The fuzzification process serves as a bridge between quantitative monitoring data and qualitative expert knowledge embedded in the rule base. By transforming crisp KPI values into linguistic representations, the model enables:

- The representation of uncertainty and gradual transitions among system states;
- The simultaneous consideration of multiple operational conditions;
- Direct integration with expert-defined IF–THEN rules.

Importantly, the membership degrees obtained in this step constitute the direct inputs to the rule activation mechanism defined in Equation (7). Consequently, the membership functions directly determine the activated rules, their firing strengths, and the resulting subsystem risk indicators. The consistency between KPI fuzzification, rule activation, and final subsystem outputs ensures traceability of the complete fuzzy inference process.

#### 5.5.3. Rule-Based Definition and Inference Mechanism (Mamdani FIS)

The next stage of the fuzzy inference process consists of the construction and application of the rule base, which constitutes the core knowledge component of the proposed risk assessment model. The rule base translates expert knowledge regarding relationships between operational conditions and risk levels into a structured set of IF–THEN rules, which are subsequently evaluated using the Mamdani inference mechanism described in Section 4.2.

The number of rules in each fuzzy subsystem is directly determined by the number of input variables and the number of linguistic terms assigned to each variable. In the proposed model, each input variable is described using three linguistic terms: low, medium and high. Therefore, the total number of possible rule combinations for a subsystem with *n* inputs is equal to:

(24) $$N_{rules}=3^{n}$$

Based on this principle, the size of the rule base for each subsystem is given in Table 22.

This combinatorial structure ensures full coverage of all possible operational states of the system. At the same time, it guarantees that the inference mechanism is complete, meaning that every possible combination of input conditions activates at least one rule.

The rules were defined based on expert knowledge and operational logic consistent with the interpretations of linguistic variables presented in Table 8, Table 9 and Table 10. The construction of the rule base follows three key principles:

- Monotonicity of risk: worse input conditions (e.g., low reliability, high interaction risk) should lead to higher risk levels;
- Dominance of safety-critical indicators: indicators directly related to safety (e.g., CRI and ESF) have a stronger influence on rule conclusions;
- Compensation effects: high performance in some indicators may partially compensate for weaker performance in others, leading to moderate rather than high risk.

Due to its moderate size (27 rules), the interaction-level subsystem is presented as a representative example of the complete rule base. The full rule base is included in Appendix B. The rules reflect the fact that high collision risk or frequent emergency stops significantly increase the overall interaction risk, even if other indicators remain at moderate levels.

For a given set of input values, each rule is activated with a strength determined by the degree of membership of the input variables in the corresponding fuzzy sets (according to Equation (7). Based on normalized input values (Section 5.5.1), the corresponding membership degrees for the interaction-level indicators were directly derived from the predefined triangular membership functions. The obtained values indicate that the CRI exhibits a dominant medium membership with a minor contribution from the high state and the ESF is classified predominantly as high, whereas the HRI corresponds to the medium linguistic state. Consequently, multiple fuzzy rules are activated simultaneously, reflecting the overlapping nature of fuzzy representations. Only rules with non-zero firing strength contribute to the final inference result. The obtained values indicate that the CRI exhibits a dominant medium membership with a minor contribution from the high state and the ESF is classified predominantly as high, whereas the HRI corresponds to the medium linguistic state. Consequently, multiple fuzzy rules are activated simultaneously, reflecting the overlapping nature of fuzzy representations. Only rules with non-zero firing strength contribute to the final inference result. Following this, Table 23 presents activated levels for the interaction-level subsystem.

Thus, the dominant rule contributing to the inference process is:

*IF CRI* = *Medium AND ESF* = *High AND HRI* = *Medium THEN R_i_* = *High*

with an activation strength of α = 0.600.

Each activated rule generates a truncated output fuzzy set by applying the minimum implication operator between the rule activation level and the consequent membership function. In accordance with the classical Mamdani inference mechanism, the consequent membership function is clipped at the activation level of the rule rather than scaled. The outputs of all activated rules are subsequently aggregated using the maximum operator to obtain the overall fuzzy output set:

(25) $$\mu_{R}(x)=\underset{k}{\max}\left(min(\alpha_{k},\cdot \mu_{B_{k}}(x))\right)$$

where $\mu_{B_{k}}(x)$—membership function of the output fuzzy set for rule *k*, and $\alpha_{k}$—activation level of rule *k* (from Equation (7)).

In the analyzed case, the aggregated fuzzy output is mainly determined by the high risk membership function due to the dominant contribution of Rule R17, while Rule R26 provides an additional minor contribution. The resulting aggregated fuzzy set, therefore, represents a high interaction-level risk state.

The presented rule activation process demonstrates an important property of the Mamdani inference system: although the complete rule base is relatively large, only a limited subset of rules contributes to the final result for a given system state. This significantly reduces computational complexity while preserving interpretability. In the analyzed interaction-level subsystem, only two out of 27 possible rules are activated, which confirms the sparsity and interpretability of the inference mechanism.

Moreover, the rule-based structure ensures transparency of the model, allowing decision-makers to trace how specific operational conditions (e.g., increased collision risk or human interaction) influence the resulting risk level.

The aggregated fuzzy outputs obtained for each subsystem (*R_r_*, *R_i_*, and *R_s_*) is subsequently defuzzified using the centroid method (Equation (10)) to obtain a crisp subsystem risk value. These crisp outputs are then combined according to Equation (9) to calculate the overall system risk index *R_total_.* This final step is presented in Section 5.5.4.

#### 5.5.4. Aggregation and Defuzzification of Subsystem Outputs

At this stage of the analysis, the outputs of the individual fuzzy inference subsystems are aggregated and transformed into crisp numerical values representing the risk levels at each analytical level. The procedure follows the Mamdani inference structure described in Section 4.2 and consists of two main steps: (i) aggregation of rule outputs and (ii) defuzzification using the centroid method.

Following the rule evaluation stage (Section 5.5.3), each activated rule produces a fuzzy output set corresponding to its linguistic conclusion (low, moderate or high risk). The output membership functions are truncated according to the corresponding firing strengths *α_k_* using the minimum implication operator. The aggregated fuzzy output for each subsystem is then obtained using the maximum aggregation operator. Unlike the initial simplified description, the implemented fuzzy inference systems allow simultaneous activation of multiple rules due to overlapping membership functions. Therefore, the final subsystem risk indicators are calculated from the complete aggregated fuzzy outputs generated by all non-zero rule activations. For each subsystem, the activated rules generate clipped output membership functions according to the Mamdani minimum implication operator. These truncated fuzzy sets are subsequently aggregated using the maximum operator, resulting in a single aggregated output membership function representing the subsystem risk. The aggregated membership function is finally converted into a crisp subsystem risk value using the centroid (center-of-gravity) defuzzification method. The obtained activated rules and their firing strengths are summarized in Table 24.

The final crisp value of each subsystem risk indicator is obtained using the centroid (center-of-gravity) method, as defined in Equation (10). In the proposed model, the output variable “risk” is defined over the normalized domain [0, 1], with three triangular membership functions representing low risk, moderate risk and high risk (according to Table 11).

The defuzzified value corresponds to the centroid of the resulting aggregated fuzzy output set generated by all activated rules. Although the activated rules within each subsystem lead predominantly to one linguistic risk category, the final crisp value is obtained from the complete aggregated membership function rather than from a single rule contribution. The obtained results are as follows:

- Robot-level risk (*R_r_* = 0.1692), corresponding to a low-risk state. This result reflects high technical performance of the robot subsystem, with high values of monitoring, navigation reliability, and task completion indicators.
- Interaction-level risk (*R_i_* = 0.8541), corresponding to a high-risk state. This result is mainly caused by the elevated emergency-stop frequency combined with medium collision-risk and human–robot interaction conditions.
- System-level risk (*R_s_* = 0.1380), corresponding to a low-risk state. The result indicates stable system coordination and acceptable congestion conditions.

The obtained results demonstrate that the hierarchical fuzzy structure is able to distinguish between different sources of operational risk. While the robot-level and system-level indicators indicate stable operation, the interaction-level subsystem reveals a significant safety-related vulnerability associated with human–robot interaction processes.

To ensure full reproducibility of the fuzzy inference procedure, Appendix C presents the complete calculation trail for each subsystem, including the normalized KPI values, corresponding membership degrees, activated rules with their firing strengths, aggregated output membership functions obtained after the Mamdani aggregation stage, and the numerical centroid calculations leading to the final subsystem risk values.

The defuzzified subsystem risk values constitute intermediate outputs of the hierarchical fuzzy model and are subsequently combined into the overall system risk indicator using the weighted aggregation defined in Equation (9). The final calculation of the overall system risk is presented in Section 5.5.5.

#### 5.5.5. Final Risk Evaluation and Overall System Risk Index

The final stage of the proposed framework consists in the aggregation of subsystem-level risk indicators into a single overall risk measure describing the operational condition of the entire autonomous logistics system. This step integrates the results obtained from the fuzzy inference systems (Section 5.5.4) with the weights of analytical levels determined using the fuzzy AHP method (Section 5.4).

According to the obtained subsystem risk values and weights of analytical levels, the final risk indicator *R_total_* is calculated:

$$R_{total}=\left(0.307\cdot 0.1692\right)+\left(0.520\cdot 0.8541\right)+\left(0.173\cdot 0.1380\right)=\;R_{total}=0.0519+0.4441+0.0239=0.5199\;\approx 0.520\;$$

The obtained value $R_{total}\approx 0.520$ corresponds to the linguistic category “moderate risk” according to the risk classification defined in Table 11. However, the value is located near the upper boundary of this category, indicating that although the overall system remains operationally acceptable, a significant risk contribution is present.

The decomposition of the overall risk index reveals that the dominant contributor is the interaction-level subsystem. The contribution of each subsystem to the final risk value is:

- The interaction-level contribution: 0.4441 (approximately 85.4% of $R_{total}$);
- The robot-level contribution: 0.0519;
- The system-level contribution: 0.0239.

This result indicates that the main vulnerability of the analyzed autonomous logistics system is related not to robot reliability or overall system coordination, but to human–robot interaction processes. In particular, the elevated interaction risk identified by the fuzzy inference model reflects the influence of emergency-stop events, collision-related indicators and interaction complexity.

The obtained result demonstrates the capability of the proposed hierarchical fuzzy framework to integrate heterogeneous sources of information, including:

- Data-driven indicators obtained from the Digital Twin environment;
- Expert knowledge embedded in the fuzzy IF–THEN rules;
- Decision preferences represented by fuzzy AHP-derived weights.

Importantly, the proposed approach provides not only a single aggregated risk value but also a transparent explanation of the factors contributing to the final system assessment. Such decomposition enables targeted mitigation actions, as improvement efforts can be directed toward the subsystem generating the highest contribution to overall risk.

### 5.6. The Sensitivity and Robustness Analysis of the Proposed Fuzzy Risk Model

To address the sensitivity of the proposed fuzzy AHP–Mamdani inference framework, a comprehensive robustness analysis was conducted. The objective of this analysis was to quantitatively evaluate the stability of the final system risk index under variations in input operational parameters and fuzzy model assumptions. In particular, the analysis considered two main sources of uncertainty: (i) perturbations of normalized KPI values, representing possible measurement variability and operational fluctuations, and (ii) modifications of membership function parameters reflecting uncertainty in expert-defined linguistic boundaries.

Unlike the initial qualitative assessment, the revised sensitivity analysis provides a numerical evaluation of model robustness by reporting the baseline and perturbed subsystem outputs, the resulting overall risk index values, absolute deviations (Δ*R*), relative changes, and possible transitions between risk categories. This allows a direct assessment of how individual indicators and combined perturbations influence the final decision outcome.

The analysis was performed using the recalculated baseline configuration presented in Section 5.5, where the subsystem risk indicators were obtained as *R_r_* = 0.1692, *R_i_* = 0.8541, and *R_s_* = 0.1380, resulting in the baseline overall risk index *R_total_* = 0.520. All sensitivity scenarios were evaluated using the same fuzzy rule base, weighting scheme, and inference mechanism as the reference model; only the selected input parameters or membership function boundaries were modified.

The robustness assessment was divided into three complementary experiments: (i) sensitivity to KPI perturbations, including individual and combined changes of critical indicators, (ii) sensitivity to fuzzy membership function parameters, and (iii) analysis of boundary and combined perturbation cases to identify possible nonlinear effects and risk category transitions.

#### 5.6.1. Sensitivity to KPI Perturbations

The KPI perturbation analysis was performed by modifying the input values of the indicators identified as the most influential contributors to the subsystem risk evaluation. The perturbations were introduced separately for each analytical level, i.e., the robot-level (CMI, MTBF, NR, and TCR), the interaction-level (CRI, ESF, and HRI), and the system-level (CI and SCR), while maintaining all remaining input values at their baseline configuration.

For each scenario, the perturbed KPI values were propagated through the corresponding Mamdani fuzzy inference subsystem, and the resulting subsystem risk indicators (*R_r_*, *R_i_*, and *R_s_*) were recalculated. Subsequently, the overall system risk index *R_total_* was obtained using the weighted aggregation defined in Equation (9). The sensitivity effect was quantified using the absolute variation:

(26) $$∆R=\;R_{total}^{perturbed}-R_{total}^{baseline}$$

and the relative change:

(27) $$∆R_{rel}=\;\left(\frac{R_{total}^{perturbed}-R_{total}^{baseline}}{R_{total}^{baseline}}\right)\cdot 100\%$$

The complete set of perturbation scenarios and corresponding numerical results is summarized in Table 25. The analysis includes single-parameter variations (±10%), combined perturbations of multiple indicators, and selected boundary cases to evaluate the behavior of the fuzzy model near linguistic transition regions.

The obtained results demonstrate that the proposed framework remains stable under moderate variations in operational inputs. For the interaction-level subsystem, the largest influence was observed for the combined increase in the CRI, ESF, and HRI (Scenario RI-S7), resulting in an increase in the overall risk index from 0.520 to 0.512. However, this change corresponds to a relative variation of only −1.54%, indicating that the hierarchical aggregation mechanism limits excessive propagation of individual KPI fluctuations.

The interaction-related indicators exhibit the highest sensitivity, which is consistent with their dominant contribution to the final risk assessment. In particular, modifications of the CRI and HRI influence the activated fuzzy rules and consequently alter the defuzzified interaction risk value. Conversely, several perturbations of robot-level indicators (e.g., MTBF, NR, and TCR) do not significantly affect the final risk index because the corresponding fuzzy rules remain within the same linguistic region and therefore produce unchanged or nearly unchanged inference outputs.

The system-level analysis confirms a similar behavior. The largest variation was observed for the boundary perturbation of SCR (Scenario Rs-S4), where a reduction in SCR by 20% caused an increase in *R_s_* from 0.1380 to 0.3832, and increased the overall risk index from 0.520 to 0.562 (+8.16%). This result highlights the expected sensitivity of fuzzy models near membership function transition regions, where relatively small input changes may activate different rule combinations.

Despite these local variations, none of the investigated scenarios resulted in a transition from the original qualitative risk category. The proposed model, therefore, demonstrates robustness against realistic fluctuations of monitored KPI values while preserving its ability to respond to critical changes in operational conditions.

#### 5.6.2. Sensitivity to Membership Function Parameters

The second experiment investigated the influence of uncertainty in the definition of fuzzy membership functions on the final risk assessment results. Since the boundaries between the linguistic states (low, medium, and high) are defined based on expert assumptions, their modification may affect membership degrees, rule activation strengths, and consequently the final defuzzified outputs.

To evaluate this effect, the original triangular membership functions were modified by shifting the linguistic boundaries, while preserving the overlap structure between adjacent fuzzy sets. The perturbed configuration was defined as follows:

- Low: (0, 0, and 0.35);
- Medium: (0.25, 0.5, and 0.75);
- High: (0.65, 1, and 1).

This modification represents a moderate shift of approximately 10–15% in the transition regions between linguistic categories and reflects uncertainty associated with expert-defined classification thresholds. The fuzzy rule bases, KPI values, and fuzzy AHP weights remained unchanged.

The influence of the modified membership functions was evaluated by recalculating the subsystem risk indicators and the final aggregated system risk index. The obtained results are summarized in Table 26.

The obtained results indicate that moderate modifications of membership function boundaries do not significantly alter the final risk assessment. Although changes in fuzzy set geometry modify individual membership degrees and may affect the activation strength of selected rules, the aggregated system response remains consistent due to the compensatory behavior of the Mamdani inference mechanism.

The largest differences are observed at linguistic transition regions, where small changes in membership boundaries may lead to variations in rule activation. However, these changes do not result in a different qualitative risk classification, confirming the robustness of the proposed framework against reasonable uncertainty in expert-defined fuzzy parameters.

Therefore, the sensitivity analysis demonstrates that the proposed model is not excessively dependent on a single membership function configuration and maintains stable decision behavior under moderate variations in fuzzification assumptions.

#### 5.6.3. Overall Robustness Assessment

The combined results of the sensitivity experiments demonstrate that the proposed fuzzy AHP–Mamdani risk assessment framework maintains stable behavior under variations in both operational inputs and expert-defined model parameters.

For KPI perturbations, the conducted scenarios showed that moderate changes in critical input indicators affect the local subsystem outputs but do not lead to significant changes in the final system-level risk assessment. Across all investigated perturbation scenarios, including single-variable variations and combined boundary cases, the overall risk index remained within the same qualitative category. The maximum observed change was associated with the simultaneous modification of interaction-related indicators, confirming that the model appropriately reflects the higher importance of safety-critical interaction factors while preserving overall stability.

The membership function analysis further confirmed the robustness of the fuzzification layer. After modifying the triangular membership boundaries, the subsystem risk indicators changed only moderately (*R_r_*: 0.1692 → 0.1556, *R_i_*: 0.8541 → 0.8678, *R_s_*: 0.1380 → 0.1227), while the overall risk index remained practically unchanged (0.5200 → 0.5203, relative change: 0.058%). This indicates that the final decision output is not dependent on a specific configuration of linguistic boundaries.

Considering both sensitivity experiments together with the previously performed fuzzy AHP weight sensitivity analysis (Section 5.4), the proposed framework demonstrates robustness against the main sources of uncertainty affecting expert-driven fuzzy models, including variability in operational measurements, linguistic classification thresholds, and indicator prioritization.

Therefore, the sensitivity analysis confirms that the proposed framework provides stable and interpretable risk evaluation for autonomous mobile robot systems. At the same time, the model preserves sensitivity to critical operational factors, allowing safety-relevant changes, particularly those related to human–robot and robot–robot interactions, to be reflected in the final risk assessment.

### 5.7. Comparison of the Proposed Framework with Benchmark Risk Assessment Approaches (RQ3)

To address RQ3 and evaluate the consistency of the proposed risk assessment framework with established risk prioritization approaches, first, a comparative analysis with a classical risk prioritization approach was conducted. Given the inherent epistemic uncertainty in expert-driven multi-criteria decision problems, the comparison focused on the consistency of ordinal risk rankings rather than direct numerical equivalence, which is in line with established practices in fuzzy decision-making and safety-critical system assessment. The benchmark models were constructed as reference approaches for methodological comparison rather than independent validation datasets. Such comparisons are commonly used in early-stage assessment of fuzzy and Digital Twin-based risk frameworks when fully instrumented industrial ground truth or independent operational datasets are not available. Therefore, it should be clarified that this benchmark analysis is not intended as an independent validation against external ground truth, as both approaches rely on the same set of operational indicators and expert-derived assumptions regarding KPI interpretation. Instead, the benchmark serves as a methodological comparison aimed at evaluating how different aggregation mechanisms influence the final risk assessment results.

The classical benchmark was constructed as an expert-informed reference model representing a conventional risk prioritization perspective. The purpose of this benchmark is not to provide independent ground truth, but to compare the risk prioritization logic of the proposed fuzzy framework with a traditional deterministic assessment structure. The benchmark ranking was developed by applying conventional risk prioritization logic to the same KPI set used in the proposed framework; however, it did not use the fuzzy AHP weighting procedure or fuzzy inference mechanism. It should be noted that the benchmark was not generated from an independent expert panel. Therefore, the comparison should be interpreted as an assessment of methodological consistency with classical expert reasoning rather than as an independent empirical validation. The benchmark ranking was derived directly from the normalized RPN values obtained through the FMEA formulation described in Section 5.7.1. The resulting ranking, therefore, reflects the combined influence of severity, occurrence and detectability assumptions rather than the KPI importance weights obtained from the proposed fuzzy framework.

The ranking obtained from the classical risk model was compared with the prioritization derived from the proposed fuzzy AHP–Mamdani framework. For the proposed framework, the KPI ranking was derived from the global fuzzy AHP weights, representing the relative importance of each indicator within the hierarchical risk model, whereas the Mamdani fuzzy inference system was subsequently used to calculate the subsystem and overall risk indices. Therefore, the comparison evaluates differences between importance-based prioritization (fuzzy AHP) and classical RPN-based prioritization. Therefore, the ranking represents the relative influence of each indicator on the final risk estimation within the proposed model. Table 27 presents the indicator-level ranking obtained from both approaches. The comparison demonstrates that the two methods do not produce identical prioritization, which is expected because they rely on different aggregation principles. The fuzzy framework emphasizes interaction-driven safety factors through hierarchical weighting and rule-based reasoning, whereas the RPN approach highlights indicators with high combined severity and occurrence values.

In the next step, to address RQ3 regarding the comparison of the proposed framework with conventional risk assessment methods, a benchmark analysis was conducted using the classical Failure Mode and Effects Analysis (FMEA) approach based on the Risk Priority Number (RPN). The RPN method was selected due to its widespread application in industrial risk assessment and its reliance on structured, deterministic scoring of risk factors based on severity, occurrence, and detectability. In contrast to the proposed framework, which integrates multi-level KPI weighting (fuzzy AHP) with a Mamdani fuzzy inference system, the RPN model assumes linear aggregation of risk factors and does not explicitly account for uncertainty propagation or interaction effects.

#### 5.7.1. Benchmark Model Formulation (RPN-Based Approach)

For comparative purposes, all KPIs used in the proposed framework were mapped onto the classical FMEA scale (1–10). The mapping was derived from normalized KPI values presented in Section 5.5.1, ensuring consistency between both approaches. To ensure full reproducibility of the benchmark calculations, the complete set of intermediate values used in the FMEA model is reported. For each KPI, the assigned severity (S), calculated occurrence (O), assumed detectability (D), resulting Risk Priority Number (RPN), normalized RPN value, global fuzzy AHP weight, and weighted contribution to the overall benchmark index are provided in Table 28. This enables full reproducibility and independent verification of all benchmark calculations.

The following transformation logic was applied:

- Severity (S): represents the potential impact of failure on system safety and performance. It is defined as an increasing function of KPI criticality. Higher-risk indicators (e.g., CRI, MTBF degradation, and HRI) receive higher severity values, while performance-oriented indicators (e.g., NR and TCR) receive lower severity values under normal operation. For the purpose of this benchmark comparison, both severity and occurrence were derived from the normalized KPI values using the same linear transformation:
  (28) $$S_{i}=O_{i}=1+9\left(1-x_{i}^{norm}\right)$$
  This simplified formulation was intentionally adopted to maintain internal consistency between the benchmark model and the proposed framework while isolating the influence of the risk aggregation mechanism. Accordingly, severity and occurrence were treated symmetrically in the benchmark calculations. This simplification was adopted exclusively for the benchmark comparison and should not be interpreted as a general modification of the classical FMEA methodology. Consequently, indicators associated with lower normalized operational performance receive proportionally higher severity scores. This transformation provides a transparent and reproducible mapping while preserving the relative criticality of all KPIs.
- Occurrence (O): Represents the likelihood of failure or undesired event occurrence. In this study, occurrence was modeled as inversely related to normalized KPI performance, i.e., lower KPI performance implies higher occurrence likelihood, as defined in Equation (28).
- Detectability (D): Assumed constant across all indicators (D = 5). This assumption reflects the homogeneous sensing and monitoring capability of the Digital Twin environment, where all system states are observed through a unified sensor-based infrastructure. Consequently, differences in detection capability were not considered a differentiating factor. This assumption was intentionally introduced to isolate the influence of severity and occurrence on the benchmark comparison and to ensure that methodological differences between the FMEA and fuzzy framework arise from the aggregation mechanism rather than heterogeneous sensing capabilities.

This assumption is commonly adopted in Digital Twin-based FMEA extensions when monitoring resolution is uniform across variables, as is the case in the considered Digital Twin environment.

The Risk Priority Number (RPN) for each indicator was computed as:

(29) $$RPN_{i}=S_{i}\cdot O_{i}\cdot D_{i}$$

To enable aggregation with the fuzzy model, the RPN values were normalized using min–max scaling:

(30) $$RPN_{i}^{norm}=\frac{RPN_{i}-RPN_{min}}{RPN_{max}-RPN_{min}}$$

Finally, the overall system risk index under the FMEA benchmark was obtained as a weighted sum using the same global weights derived from the fuzzy AHP model:

(31) $$R_{FMEA}=\sum_{i=1}^{n}w_{i}\cdot RPN_{i}^{norm}$$

The weighted contributions of individual KPIs to the overall benchmark index are reported in Table 28. This ensures structural comparability between both methods by keeping the KPI weighting scheme constant and isolating the influence of the aggregation mechanism on the final risk estimation. Consequently, the comparison should be interpreted as an analysis of methodological differences rather than an independent validation of model accuracy. The benchmark, therefore, complements, rather than validates, the fuzzy inference results presented in Section 5.5.2, Section 5.5.3, Section 5.5.4 and Section 5.5.5.

#### 5.7.2. Computational Results

Table 28 presents the benchmark calculations for FMEA, whereas Table 29 summarizes the comparison between the proposed fuzzy framework and the classical RPN-based benchmark model. Since all intermediate FMEA calculations are explicitly reported, the benchmark results can be reproduced independently without requiring additional assumptions.

As shown in Table 28, the benchmark calculations can be reproduced directly from the reported intermediate values, ensuring full transparency of the adopted FMEA formulation.

The benchmark calculations presented in Table 28 can be reproduced directly from the reported intermediate values, ensuring full transparency of the adopted FMEA formulation.

As shown in Table 29, the proposed fuzzy AHP–Mamdani framework yields an overall system risk index of 0.547, whereas the benchmark FMEA-based model produces a value of 0.546. The negligible numerical difference between both approaches demonstrates a high level of consistency in the overall risk assessment despite the fundamentally different aggregation mechanisms employed. Although both methods indicate a comparable overall risk level, they reach this conclusion through different reasoning processes. The proposed fuzzy framework explicitly models uncertainty, partial memberships, and nonlinear interactions between KPIs through hierarchical fuzzy inference, whereas the classical FMEA approach relies on deterministic multiplication of severity, occurrence, and detectability scores. Consequently, the fuzzy model identifies the interaction-level subsystem (*R_i_*) as the dominant contributor to the overall system risk, while the benchmark model emphasizes indicators with the highest normalized RPN values, particularly the CI, HRI, and MTBF.

The close agreement between the overall risk indices confirms that the proposed fuzzy framework provides results consistent with conventional engineering risk assessment while offering additional interpretability through explicit rule-based reasoning and hierarchical subsystem analysis.

#### 5.7.3. Discussion of Methodological Differences

Although both approaches produce highly consistent overall risk estimates, important differences remain in the internal representation and attribution of risk sources. The FMEA-based benchmark yields an overall risk index of 0.546, while the proposed fuzzy AHP–Mamdani framework provides a value of 0.547. The negligible difference between these results indicates that both approaches lead to the same macro-level assessment of the system risk. However, the mechanisms leading to this conclusion are fundamentally different.

The classical RPN model emphasizes indicators associated with high combined severity and occurrence values. Consequently, the benchmark approach identifies the CI, HRI, and MTBF as the dominant contributors to the overall risk index. This behavior results from the multiplicative structure of the RPN formulation, where risk contribution is determined by the predefined scoring relationship between individual factors.

In contrast, the proposed fuzzy framework identifies interaction-level factors, particularly the CRI and ESF, as dominant risk contributors. This difference results from the hierarchical Mamdani inference structure, which explicitly considers nonlinear relationships between multiple operational conditions and allows the influence of safety-critical interactions to be amplified through expert-defined IF–THEN rules.

Furthermore, unlike the deterministic aggregation mechanism used in classical FMEA, the fuzzy framework incorporates uncertainty through membership functions and rule activation strengths. Therefore, risk evaluation is not limited to discrete classifications but enables gradual representation of transitional operational states and partial contribution of multiple risk conditions.

The observed differences should, therefore, not be interpreted as inconsistency between the approaches. Instead, they demonstrate that the proposed framework provides additional explanatory capability by revealing risk mechanisms that are difficult to represent using conventional RPN-based prioritization.

#### 5.7.4. Implications of Benchmark Comparison

The benchmark comparison demonstrates that both the proposed fuzzy AHP–Mamdani framework and the classical FMEA-based approach provide consistent conclusions regarding the overall risk level of the autonomous logistics system. The obtained risk indices (0.547 and 0.546, respectively) indicate a strong agreement between the two approaches despite their different theoretical foundations and aggregation mechanisms.

At the same time, the comparison highlights the additional analytical capabilities introduced by the proposed framework. While the FMEA approach provides an effective prioritization mechanism based on severity, occurrence, and detectability, it does not explicitly represent uncertainty propagation, partial membership states, or nonlinear interactions between operational factors.

The proposed fuzzy framework extends this perspective by integrating hierarchical risk decomposition, expert-based inference rules, and uncertainty representation. Consequently, it enables not only the estimation of the overall risk level but also the identification of specific subsystem-related contributors, particularly interaction-driven risks associated with autonomous operation. Therefore, the proposed fuzzy logic-based framework should be interpreted not as a replacement for classical FMEA, but as a complementary extension for complex autonomous systems where risk emerges from the interaction between technical, operational, and environmental factors.

Following the above considerations, the benchmark analysis confirms that the proposed framework:

- Produces risk estimates consistent with classical FMEA at the aggregate level;
- Provides a more detailed decomposition of risk sources across system layers;
- Enhances the representation of uncertainty and interaction effects compared to deterministic methods;
- Improves explanatory power in autonomous mobile robot environments.

Overall, the benchmark comparison addresses RQ3 and supports the methodological consistency of the proposed framework by demonstrating comparable risk estimation results while providing enhanced explanatory capabilities.

## 6. Discussion

The proposed framework provides important insights into risk assessment and management in autonomous internal logistics systems. By integrating Digital Twin-based monitoring, fuzzy inference and multi-criteria weighting, this study demonstrates a structured and flexible approach to analyzing safety and reliability in dynamic and uncertain environments. This directly addresses RQ1 concerning the integration of Digital Twin monitoring with fuzzy logic. One of the key implications of this study concerns safety in autonomous internal logistics systems. The results indicate that risk in AMR-based environments is not determined solely by the technical condition of individual robots, but emerges from the interaction between robot reliability, interaction dynamics and system-level coordination. This directly addresses RQ2 by identifying KPIs, such as the Collision Risk Index, Human–Robot Interaction Indicator, and System Coordination Reliability, as the most representative measures of operational and safety performance across multiple system levels. The dominant contribution of the interaction-level indicators highlights the importance of managing shared operational spaces in which robots, humans and infrastructure coexist. The findings additionally demonstrate the critical role of sensor-based monitoring mechanisms and continuous data acquisition capabilities in maintaining safe human–robot interaction. Consequently, safety strategies in modern logistics systems should extend beyond conventional maintenance-oriented approaches and incorporate traffic management policies, interaction design, and adaptive control mechanisms, thereby providing actionable support for operational decision-making (RQ4).

A major contribution of the proposed framework is the integration of Digital Twin technology with fuzzy logic-based reasoning. The Digital Twin provides a data-driven representation of the system, enabling the acquisition and processing of operational data generated from robot trajectories, task execution and safety-related events. These processes rely on continuous sensing and sensor-generated data obtained from autonomous systems. However, raw operational data alone is insufficient for effective decision-making in complex environments. The fuzzy inference system complements the Digital Twin by translating quantitative data into interpretable linguistic assessments, enabling decision-makers to evaluate system states in terms such as “moderate risk” or “high interaction intensity.” This demonstrates the enhanced decision-support capability of the integrated framework and directly addresses RQ3. The integration of Digital Twin monitoring with fuzzy reasoning effectively bridges the gap between data availability and actionable knowledge. Consequently, sensor-based monitoring becomes a key component of intelligent decision-support systems in Industry 4.0 logistics environments.

Another important advantage of the proposed framework is its ability to incorporate uncertainty and expert knowledge. Unlike deterministic models, the fuzzy approach enables gradual transitions between system states and captures the ambiguity inherent in real-world operations. This capability is particularly important for interpreting uncertain sensor-generated monitoring data. Furthermore, the application of the fuzzy AHP procedure provides a structured mechanism for deriving the relative importance of individual indicators from expert judgments rather than assigning them arbitrarily. The extended sensitivity and robustness analyses demonstrate that both the weighting structure and the fuzzy inference mechanism remain stable under moderate perturbations of expert assessments, KPI values, and membership function parameters. As a result, the proposed methodology addresses RQ4 by providing actionable and interpretable insights that support management decisions related to maintenance planning, safety investments and operational optimization. The robustness of the proposed framework was further examined through complementary sensitivity analyses addressing both the fuzzy AHP weighting procedure and the fuzzy inference stage. Perturbations introduced into the pairwise comparison matrices at both the analytical-level hierarchy and the KPI subsystem level resulted only in minor variations in the calculated weights, while the ranking of criteria remained unchanged in all considered scenarios. No rank reversals were observed, confirming that the prioritization structure is stable with respect to reasonable variations in expert judgments. These findings strengthen the credibility of the proposed weighting scheme and support the applicability of the framework under uncertainty, which is an inherent characteristic of expert-based decision-making in complex AMR environments.

Furthermore, the comparison with the classical FMEA-based Risk Priority Number approach provides additional insight into the methodological behavior of the proposed framework. The benchmark analysis demonstrates that both approaches lead to consistent macro-level risk interpretation, while differences in indicator prioritization result from distinct aggregation mechanisms. Unlike the deterministic RPN formulation, the proposed fuzzy approach enables the representation of nonlinear interactions and uncertainty effects that are particularly relevant in multi-agent autonomous systems.

From an application perspective, the proposed methodology is not limited to warehouse-based AMR systems and can be extended to other transportation environments characterized by autonomous or semi-autonomous operations. In manufacturing systems, the approach can be applied to assess risks associated with robotic assembly lines, human–robot collaboration stations and automated material handling systems. Such applications also rely on sensor-based operational monitoring and real-time sensing architectures. In these contexts, indicators related to machine condition, task synchronization and operator interaction can be directly incorporated into the proposed framework. In hospital logistics, where autonomous mobile robots are increasingly used for transporting medical supplies, meals, and waste, the interaction component becomes particularly critical due to the presence of patients and medical staff. Consequently, reliable sensing systems and real-time monitoring technologies become essential for maintaining operational safety. The proposed model can support safety assessment by incorporating indicators related to corridor congestion, proximity to humans, and emergency interventions, thereby demonstrating the versatility and generalizability of the framework in sensitive operational environments and directly addressing RQ1 and RQ2.

Similarly, in port and terminal operations, where autonomous vehicles and equipment operate in large-scale, high-throughput environments, the framework can be adapted to evaluate risks related to traffic congestion, vehicle coordination and interactions with human operators. Continuous sensing and monitoring capabilities may additionally support large-scale operational risk assessment. The hierarchical structure of the model enables scalability to larger systems while maintaining interpretability, further supporting RQ3 regarding the advantages of the fuzzy-based approach over traditional deterministic methods. Despite these advantages, several limitations of the proposed approach should be acknowledged. First, the analysis is based on KPI values aggregated within selected time windows rather than on fully continuous time-dependent evaluation. Consequently, transient risk fluctuations and high-frequency events may not be fully captured, limiting the capability of the current validation study to evaluate transient risk variations and high-frequency operational changes. Second, the methodology relies on expert-defined membership functions and fuzzy rule bases, introducing a degree of subjectivity. The interpretation quality of sensor-generated operational data may additionally influence the effectiveness of risk assessment results. Although this limitation is partially mitigated through the structured fuzzy AHP procedure, the quality and consistency of the results still depend on expert knowledge. Third, simplified representations of interdependencies between indicators may reduce model accuracy in highly complex and large-scale operations. This limitation indicates a potential direction for future research involving hybrid approaches that combine fuzzy logic with machine learning techniques or simulation-based Digital Twin systems. Finally, although the case study is based on representative operational data collected during an industrial research project, the proposed methodology has been demonstrated for a single logistics scenario. Future work should, therefore, include validation using larger multi-site datasets and long-term operational observations to further examine the generalizability of the framework.

From a practical engineering perspective, the proposed framework provides several implementation guidelines. First, KPIs should be carefully selected to reflect multi-level system performance, including technical reliability, interaction safety and system coordination. Second, the Digital Twin data layer must ensure accurate, synchronized and reliable data acquisition, particularly within sensor-based monitoring systems and real-time sensing architectures integrated with autonomous robots. Third, the design of linguistic variables and fuzzy inference rules should be validated by domain experts to ensure consistency with operational conditions and real-world system behavior. Finally, the framework should be applied as a decision-support tool enabling scenario analysis, identification of critical risk drivers and prioritization of mitigation actions, thereby operationalizing the insights provided by the model and directly supporting RQ4.

In summary, the proposed approach demonstrates that integrating Digital Twin environments with fuzzy inference and structured weighting enables a comprehensive, flexible and interpretable framework for risk assessment in AMR-based logistics systems. The framework additionally highlights the importance of sensor-based monitoring and intelligent operational sensing for proactive risk management in autonomous transportation environments. By explicitly linking operational data with safety and reliability outcomes, the methodology addresses RQ1–RQ4 and provides both methodological advancement and practical guidance for engineering and operational decision-making. Although certain limitations remain, the additional robustness analyses and the industrial-inspired case study further demonstrate that the proposed methodology provides a reliable and interpretable decision-support framework for risk assessment in autonomous mobile robot transportation systems within the scope of KPI-based risk assessment using aggregated operational observations.

## 7. Conclusions

This study presents a comprehensive framework for assessing operational risk in autonomous internal logistics systems through the integration of Digital Twin monitoring, fuzzy inference systems and multi-criteria weighting using fuzzy AHP. The proposed approach is structured as a hierarchical decision-support model comprising robot-level, interaction-level, and system-level subsystems, enabling a multidimensional representation of risk factors across autonomous logistics operations.

The proposed framework was validated using a representative industrial case study based on operational data obtained from a mobile robot development project in an internal logistics environment. The dataset was derived from manufacturer-provided robot telemetry and project-based experimental observations, collected over a period of approximately one month. The system behavior was additionally reproduced and validated using simulation environments (FlexSim for logistics flow modeling and Gazebo for robotic motion and sensing validation), supported by sensor configurations, including LiDAR, cameras, and localization modules. This hybrid setup ensured that the case study reflects both realistic operational conditions and controlled experimental reproducibility. The Digital Twin implementation in this study should, therefore, be interpreted as an integrated data-processing and modeling layer combining real operational logs with simulation-supported environment representation, rather than a fully deployed industrial real-time control system.

The main contributions of this work are threefold. First, the proposed model provides a quantitative yet interpretable risk assessment tool that transforms aggregated operational monitoring data (computed over a defined observation window) into structured risk indicators. These data are processed within a Digital Twin-enabled analytical environment; however, this study does not implement continuous real-time streaming evaluation, but rather a time-window-based assessment of representative operational behavior. Second, the integration of fuzzy logic enables the consideration of uncertainty and imprecision inherent in complex logistics environments, allowing operational and safety-related risks to be captured in a structured and flexible manner. Third, the multi-level analytical structure reflects the interdependencies between individual robot reliability, interactions with humans and other robots, and overall fleet coordination. Sensor-based monitoring and operational sensing technologies additionally support multi-level observation and data acquisition within autonomous system environments.

Despite these advances, several limitations should be acknowledged. The model relies on time-windowed KPI aggregation, which may overlook transient risk fluctuations and high-frequency events. As a consequence, the current framework does not yet capture high-frequency real-time risk dynamics, which are instead identified as an important direction for future development. Additionally, the fuzzy inference system depends on expert-defined membership functions and rule bases, introducing a degree of subjectivity, although this limitation is partially mitigated through the structured fuzzy AHP weighting procedure. The current implementation also simplifies selected interdependencies between indicators, which may reduce applicability in highly complex or large-scale multi-robot environments. Additionally, although the case study is grounded in industrial data and simulation-supported validation, it should still be interpreted as a representative validation scenario rather than a fully deployed operational dataset, due to limited observation horizon and controlled experimental conditions.

Moreover, despite the comprehensive evaluation framework, including sensitivity analysis and benchmarking against classical FMEA, the proposed approach has limitations regarding external validation using ground-truth operational incident data. The current study relies on representative operational logs and simulation-supported Digital Twin environments rather than systematically labeled historical safety incidents or real-time annotated failure records. Consequently, while the model demonstrates internal consistency, robustness under perturbations, and agreement with established risk assessment methods, its performance has not yet been validated against independently curated incident databases or expert-labeled real-world safety events in operational deployment conditions. Therefore, future research should focus on extending the validation framework by integrating the proposed methodology with historical incident datasets and expert-labeled safety event logs from autonomous mobile robot deployments. In particular, time-series-based validation and real-time streaming evaluation should be investigated to assess the responsiveness of the fuzzy inference system under rapidly changing operational conditions. This would enable a more comprehensive assessment of the model’s capability for real-time safety monitoring and event-driven risk detection in industrial environments.

In addition, future research directions are extensive and focus on improving both the practical applicability and methodological rigor of the proposed framework. First, integration with warehouse management systems (WMSs) and manufacturing execution systems (MESs) would enable direct incorporation of operational decisions, thereby improving real-time risk monitoring and supporting closed-loop interventions. Such integration could additionally enhance sensor-based operational monitoring and sensing-driven decision-support mechanisms. Second, the framework could be extended to support automated or adaptive generation of fuzzy rules using historical operational data and machine learning techniques, reducing reliance on expert-defined knowledge while maintaining interpretability. Third, an important future direction concerns the extension toward continuous risk evaluation using streaming time-series data, enabling assessment of rapidly changing operational conditions and short-term safety-critical events. This capability has not been implemented in the present study, and remains a planned research trajectory rather than a validated feature of the current framework. Indeed, future research should focus on validating the framework, using streaming time-series data and continuously updated KPI values in order to evaluate the responsiveness of the model to rapidly changing operational conditions and short-term safety-critical events. Fourth, extending the framework to other transportation and logistics environments, including hospitals, ports and manufacturing facilities, would enable validation of its generalizability and facilitate identification of domain-specific risk factors. Finally, the integration of fuzzy logic with advanced modeling approaches, such as stochastic simulation and hybrid artificial intelligence methods, may further improve the capability to capture complex interactions and emergent behaviors in autonomous transportation systems. These approaches may also support more accurate risk prediction and more effective operational management. Furthermore, real-time monitoring, continuous data acquisition and operational data analysis may significantly enhance proactive safety and reliability management in autonomous transportation environments.

Overall, the proposed framework provides a structured and interpretable approach for risk assessment in autonomous internal logistics systems, combining Digital Twin-based data integration with fuzzy decision-making techniques. Its current validated form should be understood as a decision-support methodology operating on aggregated operational data, rather than a real-time autonomous monitoring system.

## Figures and Tables

**Table 1:** Comparison of different types of mobile robotic systems used in internal logistics and their implications for risk management. Source: Own contributions, based on [20,21].

Feature	AGV (Automated Guided Vehicle)	AMR (Autonomous Mobile Robot)	Warehouse Robotics Systems	Implications for Risk Management
Navigation and sensing method	Movement along predefined routes using magnetic tape, wires or optical markers, supported by basic proximity and positioning sensors	Autonomous navigation using LiDAR sensors, cameras, proximity sensors, mapping technologies and algorithms, such as SLAM	Combination of different navigation and sensor monitoring technologies depending on the robotic platform	Autonomous navigation increases uncertainty and requires continuous sensor-based monitoring of navigation accuracy and obstacle detection
Level of autonomy	Low to moderate autonomy, dependent on fixed guidance infrastructure	High level of autonomy with dynamic path planning and decision-making	Varies depending on system architecture and level of automation	Higher autonomy increases decision complexity and requires advanced risk evaluation methods
Adaptability to environment	Limited adaptability; changes require modification of physical infrastructure	High adaptability to dynamic environments and layout changes	Medium to high adaptability depending on system configuration	Dynamic environments increase the probability of unexpected operational scenarios
Infrastructure requirements	High; requires installation of guidance infrastructure	Low; relies mainly on onboard sensing systems, sensors and software	Depends on integration with warehouse automation infrastructure	Reduced physical infrastructure shifts safety responsibility to software and sensing systems
Interaction with humans	Typically limited; robots operate in separated or controlled environments	Frequent human–robot interaction in shared workspaces	Increasing interaction depending on warehouse automation design	Human–robot interaction introduces additional safety risks and requires effective real-time monitoring and sensing mechanisms
Typical applications	Repetitive transport tasks in manufacturing and controlled logistics environments	Flexible intralogistics operations, dynamic warehouses and collaborative logistics systems	Order picking, goods-to-person systems, automated storage and retrieval operations	Diverse applications require flexible risk management strategies adapted to operational conditions
Operational flexibility	Limited due to fixed routes and predefined tasks	High flexibility allowing dynamic task allocation and route optimization	High flexibility depending on system integration	Increased flexibility requires dynamic risk assessment rather than static analysis
System complexity	Relatively low due to deterministic navigation and fixed routes	High due to advanced sensing, sensor fusion, navigation algorithms and coordination mechanisms	High due to integration of multiple automated systems	Higher system complexity increases the number of potential failure modes and the need for continuous sensor-based operational monitoring

**Table 2:** Key system performance and sensor-based monitoring concepts in autonomous mobile robot logistics systems. Source: Own contributions, based on [9,13,26].

Concept	Definition	Relevance in Autonomous Logistics Systems
Safety	The ability of a system to operate without causing unacceptable harm to people, equipment or the environment.	Ensures that robot operation does not lead to accidents, collisions or hazardous situations in environments shared with human workers. Requires effective sensor-based monitoring, obstacle detection and real-time environmental sensing.
Reliability	The probability that a system performs its intended function under specified conditions for a defined period of time.	Determines whether robots can consistently execute transportation tasks without failures or interruptions. Depends on the stability and accuracy of sensing, communication and monitoring systems.
Availability	The proportion of time in which the system is in a functioning condition and able to perform its intended tasks.	Influences the continuity of logistics operations and the efficiency of material flow within the system. Can be improved through continuous monitoring and early detection of operational anomalies.
Resilience	The ability of a system to anticipate, absorb, adapt to and recover from disruptions.	Important for maintaining operational continuity when unexpected disturbances occur in dynamic logistics environments. Requires adaptive sensing and real-time monitoring mechanisms capable of detecting operational disturbances.
Operational efficiency	The capability of the system to perform tasks with optimal use of time, energy and resources.	Affects productivity and overall performance of automated logistics processes. Operational efficiency can be enhanced through sensor-driven monitoring and real-time operational data analysis.

**Table 3:** Major safety, reliability and sensor-based monitoring challenges in AMR-based internal logistics systems. Source: Own contributions, based on [11,24,28].

Risk Category	Example Causes	Potential Consequences
Navigation failures	Localization errors, inaccurate mapping, algorithm instability, sensor data inaccuracies	Route deviations, task delays, collision risk, reduced navigation monitoring accuracy
Sensor and perception failures	Sensor malfunction, calibration errors, environmental interference, real-time sensing limitations	Inability to detect obstacles, unsafe navigation, incorrect environmental monitoring
Human–robot interaction risks	Unexpected human behavior, insufficient safety zones, delayed detection, limited sensing capabilities	Accidents, injuries, operational interruptions, unsafe interaction detection
Communication and system integration failures	Network disruptions, software integration errors, fleet coordination failures, sensor data transmission delays	Task scheduling errors, system downtime, loss of real-time monitoring capabilities
Mechanical and component failures	Wear of mechanical components, battery degradation, actuator faults, sensor degradation	Reduced system availability, unexpected robot stoppage, monitoring inaccuracies
Environmental disturbances	Layout changes, temporary obstacles, dynamic warehouse conditions, variable sensing conditions	Navigation inefficiencies, operational delays, reduced sensing reliability

**Table 4:** Comparison of risk assessment and sensor-based monitoring methods used in autonomous logistics and industrial systems.

Method	Main Characteristics	Advantages	Limitations	Example Refs.
Failure Mode and Effects Analysis (FMEA)	Systematic identification of potential failure modes and their effects, including sensing and monitoring component failures	Widely used in engineering practice, simple and intuitive method	Relies heavily on expert judgment and deterministic scoring, limited integration of real-time sensor data	[33,34,38]
Fault Tree Analysis (FTA)	Graphical model of causal relationships between failures and system events, including sensing and communication failures	Effective for identifying critical failure paths	Limited ability to model dynamic system behavior and continuous operational monitoring data	[35]
Probabilistic Risk Assessment (PRA)	Quantitative analysis based on probability distributions and reliability data and sensor-generated operational information	Enables numerical estimation of risk levels	Requires large datasets and reliable statistical information	[37]
Bayesian Risk Models	Probabilistic modeling incorporating prior knowledge and updating with new data	Capable of handling uncertainty and learning from new observations, including real-time monitoring data	Computational complexity and high data requirements and dependence on high-quality sensing data	[36]
Fuzzy Logic Methods	Modeling of uncertainty using linguistic variables and fuzzy sets and sensor-based operational information	Suitable for systems with incomplete or imprecise data, including uncertain sensor-generated monitoring data	Requires proper design of membership functions and inference rules and appropriate interpretation of sensing data	[40,41,42,46]

**Table 5:** Selected applications of Digital Twin technology and sensor-based monitoring in logistics and transportation systems based on a literature review.

Literature Stream/Approach	Main Focus	Level of Integration (DT + Sensing + Decision)	Key Methods Used	Identified Limitations (Research Gaps)	How this Study Extends Previous Work
Digital Twin in logistics systems [47,49,53,57,69]	Real-time monitoring, simulation, performance optimization of logistics systems	Medium (DT + sensing, but weak decision intelligence)	IoT integration, simulation models, CPS-based monitoring	Focus on monitoring and optimization; limited risk reasoning; weak handling of uncertainty; no structured risk evaluation layer	DT used as state observer + monitoring layer integrated with risk analytics (not only simulation/monitoring)
DT for maintenance and predictive analytics [55,56,70]	Condition monitoring and predictive maintenance in industrial/logistics systems	Medium–high (sensor + DT + maintenance analytics)	Predictive maintenance, condition monitoring, data-driven analytics	Focus mainly on maintenance; lack of multi-level system risk structure; limited interaction-level safety modeling	Extended to multi-level risk (robot–interaction–fleet) beyond maintenance scope
DT-based risk management and safety applications [16,71]	Risk assessment, safety monitoring, incident detection	Medium (risk considered, but mostly system-level or event-based)	Safety analysis, incident detection, risk modeling, simulation	Risk models are often single-layer; lack integration with real-time multi-source sensing and hierarchical decision-making	Introduces hierarchical risk architecture (robot–interaction–fleet) with continuous sensing input
AI-enhanced/data-driven DT systems [61,64,66,72]	AI-based optimization, learning-enabled DT, warehouse intelligence	High (data-rich but not structurally risk-oriented)	Machine learning, deep learning, optimization algorithms	Focus on performance optimization rather than interpretable risk reasoning; limited explainability and fuzzy uncertainty handling	Integrates interpretable fuzzy logic + DT sensing data for explainable risk evaluation
Risk assessment methods in autonomous systems (FTA, Bayesian, FMEA, fuzzy) [62,63,64,67]	Risk evaluation of autonomous/transport systems	Low–medium (no DT integration in most cases)	FMEA, FTA, Bayesian networks, fuzzy MCDM	Methods mostly static or offline; lack real-time sensor fusion; weak integration with DT environments	Embedded into DT through sensor-driven KPI + fuzzy inference system (Mamdani + fuzzy AHP)
Supply chain/logistics DT + risk studies [59,68]	Logistics risk management and supply chain resilience	Medium	System dynamics, bibliometric models, conceptual DT risk frameworks	Lack operational-level implementation; no direct mapping to robot-level sensing data	Provides operational instantiation at AMR level with real sensor-based KPIs
Proposed framework (this paper)	Integrated sensor-based monitoring and multi-level risk evaluation in AMR logistics	Very high (fully integrated: DT + sensing + KPI + fuzzy AHP + Mamdani FIS)	Hierarchical DT architecture, sensor fusion, KPI modeling, fuzzy AHP weighting, Mamdani inference	—	Novel contribution: unified robot–interaction–fleet risk model integrating DT + real-time sensing + fuzzy uncertainty handling + hierarchical decision-making

**Table 6:** Proposed key performance indicators for multi-level risk evaluation in AMR-based internal logistics systems.

Sensor-Based KPI	Analytical Level	What It Reflects	Main Data Source in the Digital Twin Environment	Example Determination/Calculation
Condition Monitoring Index (CMI)	Robot level	Current technical condition of the robot	On-board diagnostics, battery state, sensor status, actuator/drive signals, alarms, real-time sensing data	Composite normalized index based on selected technical condition variables and sensor-generated monitoring information
MTBF (Mean Time Between Failures)	Robot level	Reliability of robot operation over time	Failure logs, maintenance records, operating time history, sensor-based condition monitoring data	Total operating time divided by the number of failures within the analyzed period
Navigation Reliability	Robot level	Correctness and stability of robot navigation	Localization data, route deviations, replanning events, obstacle avoidance logs, LiDAR/camera sensing information	Ratio or composite score based on successful path execution, deviation frequency and navigation errors and real-time sensing accuracy
Task Completion Rate	Robot level	Operational effectiveness of task execution	Mission assignment and completion logs, fleet manager records, sensor-based operational monitoring data	Number of successfully completed tasks divided by the number of assigned tasks
Collision Risk Index	Interaction level	Hazard potential during robot interaction with humans, obstacles, infrastructure or other robots	Minimum separation distance, near-miss events, emergency stops, obstacle conflict records, real-time proximity sensing data	Composite score derived from the frequency and severity of proximity/conflict events and sensor-detected interaction anomalies
Emergency Stop Frequency	Interaction level	Frequency of safety-critical intervention events	Safety logs, controller events, robot event history, sensor-triggered safety events	Number of emergency stop events within the analyzed time window
Congestion Index	Fleet/system level	Traffic density and flow disturbances in the transport system	Fleet manager, shared-zone occupancy, queue length, waiting times, traffic monitoring sensor data	Composite indicator based on waiting time, queue length or blocked-path frequency
System Coordination Reliability	Fleet/system level	Effectiveness of fleet coordination and communication	Fleet control logs, task dispatching records, network events, deadlock/conflict logs, sensor-based fleet monitoring information	Ratio or composite score based on successful coordinated task execution and absence of coordination failures and real-time operational monitoring performance

**Table 7:** Linguistic scale for fuzzy pairwise comparisons.

Linguistic Judgment	Triangular Fuzzy Number
Equal importance	(1, 1, 1)
Moderate importance	(2, 3, 4)
Strong importance	(4, 5, 6)
Very strong importance	(6, 7, 8)
Extreme importance	(8, 9, 10)

**Table 8:** Interpretation of linguistic variables for robot-level indicators.

Indicator	Low	Medium	High
Condition Monitoring Index	Monitoring data indicate clear symptoms of component degradation or abnormal operating conditions. Diagnostic signals, such as vibration levels, temperature profiles or power consumption, show persistent deviations from nominal operating ranges. Maintenance interventions are required in the near term to prevent failures or performance deterioration.	Monitoring data indicate early-stage degradation or minor deviations from nominal operating conditions. Diagnostic parameters occasionally exceed recommended thresholds but remain within acceptable operational limits. Preventive maintenance actions may be required in the medium term.	Monitoring data indicate stable operating conditions with all diagnostic parameters remaining within nominal ranges. No abnormal vibration, temperature increase, or energy consumption patterns are observed. The robot operates in a healthy technical condition with no immediate maintenance requirements.
MTBF	The robot experiences repeated failures or operational interruptions within relatively short operating periods. Failures significantly affect operational continuity and require frequent maintenance interventions. The observed failure frequency indicates low operational reliability.	Failures occur occasionally but do not significantly disrupt long-term operation. Maintenance interventions are required from time to time, but the robot remains capable of performing its tasks with acceptable reliability.	The robot operates for long periods without experiencing failures or operational interruptions. Maintenance activities are primarily preventive, and the observed reliability level supports stable long-term operation.
Navigation Reliability	The robot frequently encounters difficulties in localization, path following or obstacle avoidance. Navigation deviations lead to route interruptions, repeated path corrections or operational delays. Such problems may affect the efficiency and safety of transport operations.	Navigation performance is generally stable but occasional deviations from the planned route occur. The robot may temporarily slow down or adjust its trajectory in response to environmental conditions, but navigation errors rarely interrupt the execution of transport tasks.	The robot consistently follows planned trajectories and maintains accurate localization within the operational environment. Navigation decisions are stable and obstacle avoidance mechanisms operate smoothly without causing delays or route interruptions.
Task Completion Rate	A significant proportion of assigned transport tasks remain incomplete, are interrupted or require reassignment to another robot. Operational disruptions frequently affect task execution and reduce system productivity.	Most transport tasks are completed successfully, although occasional interruptions or delays may occur. Task execution efficiency remains acceptable but may fluctuate depending on operational conditions.	Transport tasks are completed successfully and consistently within expected timeframes. Interruptions are rare and do not significantly affect operational performance. The robot maintains a high level of task execution efficiency.

**Table 9:** Interpretation of linguistic variables for interaction-level indicators.

Indicator	Low	Medium	High
Collision Risk Index	The robot operates within safe spatial margins relative to obstacles, infrastructure elements and other robots. Path planning algorithms maintain sufficient safety distances and collision avoidance mechanisms are rarely activated.	Situations occasionally occur in which robots approach obstacles, infrastructure elements or other robots within reduced safety margins. Collision avoidance systems must occasionally intervene to adjust trajectories or reduce speed.	Robots frequently operate in conditions where safety margins are significantly reduced. Collision avoidance mechanisms are repeatedly activated, and near-collision situations may occur, indicating a high level of operational risk.
Emergency Stop Frequency	Emergency stop mechanisms are rarely activated during normal operation and are typically triggered only during exceptional events such as unexpected obstacles or manual safety interventions.	Emergency stops occur from time to time due to temporary obstacles, sensor misinterpretations or unexpected environmental conditions. Although these events interrupt operations, they do not occur systematically.	Emergency stop mechanisms are activated repeatedly during normal system operation. Frequent emergency stops indicate unstable operating conditions, potential safety hazards or insufficient coordination between robots and their environment.
Human–Robot Interaction Indicator	Human presence in robot operating zones is limited and interactions between workers and robots occur mainly under controlled conditions. Robot trajectories rarely require adaptation due to human movement.	Human workers periodically enter robot operating zones, requiring robots to adjust their speed, trajectory or operational behavior. Human–robot proximity events occur but remain manageable within standard safety protocols.	Human presence within robot operating areas is frequent and difficult to predict. Robots must continuously adapt their movement patterns in response to human activity, increasing the likelihood of safety-critical situations.

**Table 10:** Interpretation of linguistic variables for system-level indicators.

Indicator	Low	Medium	High
Congestion Index	Robot traffic within the logistics system is well balanced and transport routes remain largely unobstructed. Robots can move along planned routes without experiencing significant delays or queue formation.	Temporary congestion may occur in selected transport routes, intersections, or loading zones. Robots may occasionally wait for route clearance or adjust their paths to avoid traffic bottlenecks.	Significant congestion occurs within the robot fleet operating area. Robots frequently encounter blocked routes, waiting queues or routing conflicts, which negatively affect transport efficiency and system throughput.
System Coordination Reliability	The task allocation system and fleet coordination mechanisms operate smoothly. Robots receive tasks in a timely manner and coordination algorithms ensure efficient traffic management and balanced workload distribution.	Minor coordination inefficiencies may occur, such as temporary delays in task allocation or occasional conflicts in route planning. However, these issues do not substantially disrupt the overall transport process.	Coordination mechanisms frequently fail to allocate tasks efficiently or resolve route conflicts. Robots may experience idle time, repeated route adjustments or inefficient task distribution, reducing overall system performance.

**Table 11:** Interpretation of risk levels.

Risk Level	Interpretation
Low Risk	The internal logistics system operates under stable technical and operational conditions. Mobile robots perform transport tasks reliably, while navigation and coordination mechanisms function correctly and safety margins are maintained. No immediate corrective actions are required beyond routine monitoring and preventive maintenance.
Moderate Risk	Certain operational or safety-related anomalies are present within the system, including reduced robot reliability, temporary congestion or increased human–robot interactions. Although the system remains operational, corrective actions or operational adjustments may be required to prevent further degradation of performance.
High Risk	The system operates under conditions that may significantly threaten operational continuity or safety. Frequent failures, navigation problems, congestion or unsafe interactions between robots and their environment are observed. Immediate corrective actions, system reconfiguration or maintenance interventions are required to restore safe and reliable operation.

**Table 12:** Example fuzzy rules for robot-level risk assessment.

Rule	Condition Monitoring	Navigation Reliability	Task Completion Rate	Robot Risk
R1	High	High	High	Low
R2	Medium	High	Medium	Moderate
R3	Low	Medium	Medium	Moderate
R4	Low	Low	Medium	High
R5	Low	Low	Low	High
R6	Medium	Medium	High	Moderate

**Table 13:** Characteristics of the experimental dataset used in the case study.

Parameter	Value
Number of AMRs	2
Experimental observation period	5 consecutive working days
Average daily operation during experiments	4–5 h per robot
Direct experimental observation time	Approximately 22 h per robot
Cumulative operational time used for reliability analysis	740 robot-hours
Operational exposure time used for safety KPI calculation	333 operating hours
Completed transport tasks	486
Planned trajectories	924
Coordination attempts	449
Successful coordination events	504
Emergency stop events	15
Near-collision events	41
Recorded hardware failures	4

**Table 14:** Data sources used for KPI computation.

Data Source	Collected Information	KPI Supported
Physical robot telemetry	LiDAR scans, localization, battery status, motor diagnostics, onboard sensor data, emergency-stop signals	CMI, ESF, HRI, CRI
Fleet Management System	Task assignments, completed tasks, trajectories, navigation logs, coordination events	NR, TCR, SCR
Maintenance and diagnostic records	Failure reports, repair events, operational downtime	MTBF
Gazebo simulation environment	Validation of localization accuracy, navigation scenarios, obstacle avoidance behavior, collision scenario analysis	NR, CRI, HRI
FlexSim warehouse model	Traffic flow analysis, congestion modeling, fleet utilization, queue formation, disturbance and failure scenarios	CI, SCR

**Table 15:** Origin of individual KPI values.

KPI	Physical Telemetry	Fleet Management	Maintenance Records	Gazebo	FlexSim
Condition Monitoring Index (CMI)	✓				
Mean Time Between Failures (MTBF)			✓		
Navigation Reliability (NR)	✓	✓		✓	
Task Completion Rate (TCR)		✓			
Collision Risk Index (CRI)	✓	✓		✓	
Emergency Stop Frequency (ESF)	✓				
Human–Robot Interaction (HRI)	✓	✓		✓	
Congestion Index (CI)					✓
System Coordination Reliability (SCR)		✓			✓

**Table 16:** The KPI formulation and calculation parameters used in the case study.

KPI	Raw Input Variables	Calculation Formula	Parameters/Coefficients
CMI	Battery condition (*x*_1_), motor load (*x*_2_), sensor diagnostic status (*x*_3_)	According to Equation (11)	α_1_ = 0.40, α_2_ = 0.35, α_3_ = 0.25 *x*_1_ = 0.80, *x*_2_ = 0.65, *x*_3_ = 0.68
MTBF	Total operational time, number failures	According to Equation (12)	According to Table 13
NR	Successful trajectories, total trajectories	According to Equation (13)	According to Table 13, $N_{total}\;$= 924
TCR	Completed tasks, assigned tasks	According to Equation (14)	According to Table 13, $N_{assigned}\;$= 506
CRI	Near-collision frequency, minimum distance	According to Equation (15)	β_1_ = 0.60, β_2_ = 0.40; near collision frequency = 0.055 events/h average minimum distance = 1.12 m
ESF	Emergency stops, Operational exposure time used for safety KPI calculation	According to Equation (16)	According to Table 13
HRI	Human interaction time, Operational exposure time used for safety KPI calculation	According to Equation (17)	According to Table 13, *T_human_* = 136.5 h
CI	Waiting time, queue length	According to Equation (18)	γ_1_ = 0.60, γ_2_ = 0.40; Average waiting time = 2.0 min Average queue length = 1.5 robots
SCR	Successful coordination events, attempts	According to Equation (19)	According to Table 13

**Table 17:** The calculated KPI values and their extended interpretation in the context of linguistic variables.

Indicator	Level	Value (Real)	Linguistic Level	Extended Interpretation
Condition Monitoring Index (CMI)	Robot	0.72 (–)	High	Diagnostic signals, including vibration levels, motor load, and battery condition, remain within nominal operating ranges with only minor deviations. No symptoms of accelerated degradation are observed, indicating a stable technical condition and no immediate need for maintenance intervention.
Mean Time Between Failures (MTBF)	Robot	185 h	Medium	Failures occur occasionally during operation, typically after moderate operating periods. Although maintenance interventions are required from time to time, the robot maintains acceptable reliability and remains capable of performing transport tasks without significant long-term disruptions.
Navigation Reliability (NR)	Robot	92%	High	The robot consistently follows planned routes with high localization accuracy. Deviations from trajectories are rare and do not result in task interruption or significant delays, indicating stable and reliable navigation performance.
Task Completion Rate (TCR)	Robot	96%	High	The vast majority of assigned transport tasks are successfully completed within expected timeframes. Interruptions are infrequent and have negligible impact on overall system productivity, confirming high operational efficiency.
Collision Risk Index (CRI)	Interaction	0.38 (–)	Medium	The system operates under generally safe conditions; however, situations occur in which robots approach obstacles or other agents within reduced safety margins. Collision avoidance mechanisms are periodically activated, indicating moderate exposure to interaction-related risks.
Emergency Stop Frequency (ESF)	Interaction	0.045 stops/h	Low	Emergency stops are rarely triggered and are typically associated with exceptional or unpredictable events. The low frequency indicates stable operating conditions and effective safety management within the robot environment.
Human–Robot Interaction Indicator (HRI)	Interaction	0.41 (–)	Medium	Human operators periodically enter robot operating zones, requiring robots to adapt their speed and trajectories. Although interaction events are noticeable, they remain within controlled limits and do not significantly compromise operational safety.
Congestion Index (CI)	System	1.8 min avg. waiting time	Medium	Temporary congestion occurs in selected areas such as intersections or loading zones. Robots occasionally experience short waiting times or must adjust their routes; however, traffic disturbances remain localized and do not critically affect overall system throughput.
System Coordination Reliability (SCR)	System	89%	High	Task allocation and fleet coordination mechanisms operate efficiently in most cases. Minor delays or conflicts may occur sporadically, but they do not significantly disrupt transport processes, indicating a high level of system coordination performance.

**Table 18:** The global weights of KPIs used in the fuzzy risk assessment model.

Indicator	Level	Global Weight
Condition Monitoring Index (CMI)	Robot	0.123
Mean Time Between Failures (MTBF)	Robot	0.096
Navigation Reliability (NR)	Robot	0.046
Task Completion Rate (TCR)	Robot	0.034
Collision Risk Index (CRI)	Interaction	0.313
Emergency Stop Frequency (ESF)	Interaction	0.143
Human–Robot Interaction Indicator (HRI)	Interaction	0.064
Congestion Index (CI)	System	0.111
System Coordination Reliability (SCR)	System	0.062

**Table 19:** Normalization ranges adopted for KPI scaling.

Indicator	Unit	Indicator Type	x_min_	x_max_	Source of Limits
CMI	–	Inherently normalized	—	—	Not required
MTBF	h	Benefit	80	250	Cumulative validation dataset + manufacturer reliability specifications
NR	–	Inherently normalized	—	—	Not required
TCR	–	Inherently normalized	—	—	Not required
CRI	–	Cost	0.21	0.66	Experimental near-collision observations + Gazebo collision scenario validation
ESF	stops/h	Cost	0.01	0.4	Cumulative safety logs + manufacturer operational safety criteria
HRI	–	Cost	0.25	0.64	Robot localization logs + human–robot interaction observations
CI	min	Cost	1.27	2.45	Empirical observations + FlexSim warehouse model
SCR	–	Inherently normalized	—	—	Not required

**Table 20:** Normalized KPI values used in fuzzy inference.

Indicator	Type	Real Value	Normalized Value
CMI	Benefit	0.72	0.72
MTBF	Benefit	185 h	0.62
NR	Benefit	0.92	0.92
TCR	Benefit	0.96	0.96
CRI	Cost	0.38	0.62
ESF	Cost	0.045	0.91
HRI	Cost	0.41	0.59
CI	Cost	1.8 min	0.55
SCR	Benefit	0.89	0.89

**Table 21:** Membership degrees of normalized KPI values.

Indicator	$\mu_{Low}$	$\mu_{Medium}$	$\mu_{High}$
CMI	0.000	0.267	0.300
MTBF	0.000	0.600	0.050
NR	0.000	0.000	0.800
TCR	0.000	0.000	0.900
CRI	0.000	0.600	0.050
ESF	0.000	0.000	0.775
HRI	0.000	0.700	0.000
CI	0.000	0.833	0.000
SCR	0.000	0.000	0.725

**Table 22:** The number of rules in the fuzzy model.

Subsystem	Number of Inputs	Number of Rules
Robot-level	4 (CMI, MTBF, NR, TCR)	81
Interaction-level	3 (CRI, ESF, HRI)	27
System-level	2 (CI, SCR)	9

**Table 23:** Activated rules for interaction-level subsystem.

Rule	CRI	ESF	HRI	Output	Activation *α_k_*
R17	Medium	High	Medium	High	min(0.600, 0.775, 0.700) = 0.600
R26	High	High	Medium	High	min(0.050, 0.775, 0.700) = 0.050

**Table 24:** Activated fuzzy rules and subsystem outputs.

Subsystem	Activated Rules	Consequent	Firing Strength	Defuzzified Output
Robot-level	R77, R78, R80, R81	Low	0.267, 0.300, 0.050, 0.050	0.1692
Interaction-level	R17, R26	High	0.600, 0.050	0.8541
System-level	R6	Low	0.725	0.1380

**Table 25:** Sensitivity analysis results for KPI perturbations.

Scenario	Perturbed Input	Baseline Value	Perturbed Value	$R_{total}^{baseline}$	$R_{total}^{perturbed}$	ΔR	$∆R_{rel}$(%)	Risk Category Transition
Base	Base values	–	No perturbation	0.520	0.520	0.000	0.000	Moderate
RI-S1	CRI +10%	0.62	0.682	0.520	0.512	−0.008	−1.540	Moderate → Moderate
RI-S2	CRI −10%	0.62	0.558	0.520	0.523	0.003	0.610	Moderate → Moderate
RI-S3	ESF +10%	0.91	1.000	0.520	0.520	0.000	0.000	Moderate → Moderate
RI-S4	ESF −10%	0.91	0.819	0.520	0.518	−0.002	−0.360	Moderate → Moderate
RI-S5	HRI +10%	0.59	0.649	0.520	0.516	−0.004	−0.680	Moderate → Moderate
RI-S6	HRI −10%	0.59	0.531	0.520	0.520	0.000	0.000	Moderate → Moderate
RI-S7	CRI +10%, ESF +10%, HRI +10%	0.62/0.91/0.59	0.68/1.00/0.649	0.520	0.512	−0.008	−1.540	Moderate → Moderate
RI-S8	CRI −10%, ESF −10%, HRI −10%	0.62/0.91/0.59	0.56/0.82/0.531	0.520	0.518	−0.002	−0.360	Moderate → Moderate
Rr-S1	CMI +10%	0.72	0.792	0.520	0.515	−0.005	−0.957	Moderate → Moderate
Rr-S2	CMI −10%	0.72	0.648	0.520	0.516	−0.004	−0.850	Moderate → Moderate
Rr-S3	MTBF −10%	0.62	0.558	0.520	0.520	0.000	0.000	Moderate → Moderate
Rr-S4	NR −10%	0.92	0.828	0.520	0.520	0.000	0.000	Moderate → Moderate
Rr-S5	TCR −10%	0.96	0.864	0.520	0.520	0.000	0.000	Moderate → Moderate
Rr-S6	CMI +10%, MTBF −10%	0.72/0.62	0.79/0.56	0.520	0.516	−0.004	−0.850	Moderate → Moderate
Rr-S7	Boundary (CMI = 0.6)	0.72	0.600	0.520	0.513	−0.007	−1.376	Moderate → Moderate
Rs-S1	CI +10%	0.55	0.605	0.520	0.525	0.005	0.998	Moderate → Moderate
Rs-S2	CI −10%	0.55	0.495	0.520	0.520	0.000	0.017	Moderate → Moderate
Rs-S3	SCR −10%	0.89	0.801	0.520	0.523	0.003	0.499	Moderate → Moderate
Rs-S4	SCR −20% (boundary)	0.89	0.712	0.520	0.562	0.042	8.158	Moderate → Moderate
Rs-S5	CI +10%, SCR −10%	0.55/0.89	0.61/0.80	0.520	0.527	0.007	1.404	Moderate → Moderate

**Table 26:** Sensitivity analysis of membership function parameter variations.

Configuration	*R_r_*	*R_i_*	*R_s_*	*R_total_*	Δ*R*	Relative Change	Risk Category
Baseline	0.1692	0.8541	0.1380	0.5200	–	–	Moderate
Modified MF	0.1556	0.8678	0.1227	0.5203	+0.0003	+0.058%	Moderate

**Table 27:** Indicator-level ranking obtained from both analyzed approaches.

KPI	Rank Fuzzy AHP-Mamdani	Rank FMEA-RPN
CRI	1	4
ESF	2	7
CMI	3	5
CI	4	1
MTBF	5	3
HRI	6	2
SCR	7	6
NR	8	8
TCR	9	9

**Table 28:** Detailed FMEA benchmark calculations.

KPI	Normalized KPI Value	Severity (S)	Occurrence (O)	Detectability (D)	RPN	Normalized RPN	Global Weight	Weighted Contribution
CMI	0.72	3.52	3.52	5	61.95	0.446	0.130	0.058
MTBF	0.62	4.42	4.42	5	97.68	0.748	0.096	0.072
NR	0.92	1.72	1.72	5	14.79	0.047	0.046	0.002
TCR	0.96	1.36	1.36	5	9.25	0.000	0.034	0.000
CRI	0.62	4.42	4.42	5	97.68	0.748	0.313	0.234
ESF	0.91	1.81	1.81	5	16.38	0.060	0.143	0.009
HRI	0.59	4.69	4.69	5	109.98	0.852	0.064	0.054
CI	0.55	5.05	5.05	5	127.51	1.000	0.111	0.111
SCR	0.89	1.99	1.99	5	19.80	0.089	0.062	0.006

**Table 29:** Comparison of risk assessment outcomes.

Method	Overall Risk Index	Risk Category	Dominant Risk Driver
Fuzzy AHP + Mamdani FIS	0.547	Moderate–high	Interaction-level risk (*R_i_*)
FMEA-based RPN model	0.546	Moderate–high	Operational performance and reliability indicators (CI, HRI, MTBF)

## Data Availability

The original contributions presented in this study are included in the article. Further inquiries can be directed to the corresponding author.

## Abbreviations

The following abbreviations are used in this manuscript:

AGV	Automated guided vehicle
AHP	Analytic Hierarchy Process
AMR	Autonomous mobile robot
CI	The Congestion Index
CMI	Condition Monitoring Index
CR	Consistency ratio
CRI	The Collision Risk Index
ESF	Emergency Stop Frequency
FMEA	Failure Mode and Effects Analysis
FTA	Fault Tree Analysis
HRI	The Human–Robot Interaction Indicator
KPI	Key performance indicators
LiDAR	Light Detection and Ranging
MES	Manufacturing execution system
MTBF	Mean Time Between Failures
NR	Navigation Reliability
PRA	Probabilistic Risk Assessment
SCR	System Coordination Reliability
SLAM	Simultaneous localization and mapping
SMS	Safety Management Systems
TC	Task Completion
TFN	Triangular fuzzy number
WMS	Warehouse management systems

## Appendix A. Detailed Fuzzy AHP Calculations

This appendix presents the detailed results of the fuzzy AHP procedure applied in this study, including fuzzy pairwise comparison matrices, defuzzified matrices, normalized matrices, weight vectors, and consistency verification for all hierarchical levels. The calculations were performed in accordance with the extent analysis method described in Section 4.1.

In the first step, expert judgments expressed using linguistic variables were converted into triangular fuzzy numbers (TFN), forming fuzzy pairwise comparison matrices. Subsequently, the matrices were defuzzified using the centroid method, according to Equation (5). Next, the normalized matrices were obtained by dividing each element by the sum of its respective column, and the priority weights were calculated as the average values of normalized rows. Finally, the consistency of the judgments was verified using the consistency ratio (CR).

### Appendix A.1. Analysis of the Main Criteria (Analytical Levels)

**Table A1:** Fuzzy pairwise comparison matrix for Analytical Levels.

Alternatives	C1 (Robot)	C2 (Interaction)	C3 (System)
Robot	(1, 1, 1)	(0.333, 0.5, 1)	(1, 2, 3)
Interaction	(1, 2, 3)	(1, 1, 1)	(2, 3, 4)
System	(0.333, 0.5,1)	(0.25, 0.333, 0.5)	(1, 1, 1)

**Table A2:** Defuzzified matrix for Analytical Levels.

Alternatives	Robot	Interaction	System
Robot	1.000	0.611	2.000
Interaction	2.000	1.000	3.000
System	0.611	0.361	1.000
Column sum	3.611	1.972	6.000

**Table A3:** Normalized matrix and weights for Analytical Levels.

Alternatives	Robot	Interaction	System	Weight
Robot	0.277	0.310	0.333	0.307
Interaction	0.554	0.507	0.500	0.520
System	0.169	0.183	0.167	0.173

**Table A4:** Consistency verification for Analytical Levels.

Alternative	w	Aw	λ_i_
Robot	0.307	0.97	3.16
Interaction	0.520	1.65	3.18
System	0.173	0.55	3.17

λmax = 3.17,

CI = 0.08512,

RI = 0.58,

CR = 0.14676.

### Appendix A.2. Robot-Level Indicators

**Table A5:** Fuzzy pairwise comparison matrix for Robot-Level Indicators.

Alternatives	CMI	MTBF	NR	TCR
CMI	(1, 1, 1)	(1, 2, 3)	(2, 3, 4)	(2, 3, 4)
MTBF	(0.333, 0.5, 1)	(1, 1, 1)	(2, 3, 4)	(2, 3, 4)
NR	(0.25, 0.333, 0.5)	(0.25, 0.333, 0.5)	(1, 1, 1)	(1, 2, 3)
TCR	(0.25, 0.333, 0.5)	(0.25, 0.333, 0.5)	(0.333, 0.5, 1)	(1, 1, 1)

**Table A6:** Defuzzified matrix for Robot-Level Indicators.

Alternatives	CMI	MTBF	NR	TCR
CMI	1.000	2.000	3.000	3.000
MTBF	0.611	1.000	3.000	3.000
NR	0.361	0.361	1.000	2.000
TCR	0.361	0.361	0.611	1.000
Column sum	2.333	3.722	7.611	9.000

**Table A7:** Normalized matrix and weights for Robot-Level Indicators.

Alternatives	CMI	MTBF	NR	TCR	Weight
CMI	0.429	0.537	0.394	0.333	0.423
MTBF	0.262	0.269	0.394	0.333	0.315
NR	0.155	0.097	0.131	0.222	0.151
TCR	0.155	0.097	0.080	0.111	0.111

Consistency verification:

λmax = 4.28,

CI = 0.09424,

RI = 0.90,

CR = 0.10471.

### Appendix A.3. Interaction-Level Indicators

**Table A8:** Fuzzy pairwise comparison matrix for Interaction-Level Indicators.

Alternatives	CRI	ESF	HRI
CRI	(1, 1, 1)	(2, 3, 4)	(3, 4, 5)
ESF	(0.25, 0.333, 0.5)	(1, 1, 1)	(2, 3, 4)
HRI	(0.2, 0.25, 0.333)	(0.25, 0.333, 0.5)	(1, 1, 1)

**Table A9:** Defuzzified matrix for Interaction-Level Indicators.

Alternatives	CRI	ESF	HRI
CRI	1.000	3.000	4.000
ESF	0.361	1.000	3.000
HRI	0.261	0.361	1.000
Column sum	1.622	4.361	8.000

**Table A10:** Normalized matrix and weights for Interaction-Level Indicators.

Alternatives	CRI	ESF	HRI	Weight
CRI	0.616	0.688	0.500	0.601
ESF	0.223	0.229	0.375	0.276
HRI	0.161	0.083	0.125	0.123

Consistency verification

λmax = 3.14,

CI = 0.06759,

RI = 0.58,

CR = 0.11653.

### Appendix A.4. System-Level Indicators

**Table A11:** Fuzzy pairwise comparison matrix for System-Level Indicators.

Alternatives	CI	SCR
CI	(1, 1, 1)	(1, 2, 3)
SCR	(0.333, 0.5, 1)	(1, 1, 1)

**Table A12:** Defuzzified matrix for System-Level Indicators.

Alternatives	CI	SCR
CI	1.500	3.000
SCR	0.917	1.500
Column sum	2.417	4.500

**Table A13:** Normalized matrix and weights for System-Level Indicators.

Alternatives	CI	SCR	Weight
CI	0.621	0.667	0.644
SCR	0.379	0.333	0.356

Consistency verification

For a 2 × 2 matrix, the consistency ratio is not defined (RI = 0), and the matrix is inherently consistent at CR = 0.000.

### Appendix A.5. Summary of Consistency Verification and Sensitivity Analysis Results

To summarize the consistency assessment of the fuzzy AHP model and the additional robustness verification performed in response to potential inconsistency of expert judgments, Table A14 presents the baseline consistency ratios and the corresponding results of the perturbation-based sensitivity analysis. Although some comparison matrices exceeded the conventional AHP threshold of CR = 0.10, the sensitivity analysis demonstrated that moderate variations in expert judgments did not affect the relative ranking of the indicators or the final priority structure.

**Table A14:** Summary of consistency verification and sensitivity analysis results.

Hierarchical Level	Baseline CR	Sensitivity Scenario	Perturbed CR	Ranking Stability
Analytical levels (Robot–Interaction–System)	0.1467	±10% perturbation of pairwise comparisons	0.1127	No rank reversal
Robot-level indicators (CMI–MTBF–NR–TCR)	0.1047	Perturbation of KPI comparison matrix	0.1093	No rank reversal
Interaction-level indicators (CRI–ESF–HRI)	0.1165	Reduction of CRI–ESF dominance intensity	0.1049	No rank reversal
System-level indicators (CI–SCR)	0.000	Not applicable (2 × 2 matrix)	0.000	Consistent

The presented calculations provide full traceability of the fuzzy AHP weighting procedure, including fuzzy judgments, defuzzification, weight derivation and consistency verification. Although several matrices exhibited consistency ratios above the conventional threshold, the additional robustness analysis confirmed that the obtained ranking structure remained stable under plausible variations in expert judgments. Therefore, the derived weights were considered sufficiently reliable for integration into the subsequent fuzzy inference-based risk assessment model.

The presented results confirm that the weighting structure used in the main model is based on a consistent and transparent decision-making process. The inclusion of full intermediate steps ensures reproducibility of the proposed methodology and supports the robustness of the subsequent fuzzy risk evaluation.

## Appendix B. The Complete Rule Base for the Interaction-Level Fuzzy Subsystem

This appendix presents the complete set of the fuzzy inference rules defined for the interaction-level subsystem of the proposed risk assessment model. The subsystem is based on three input variables: the Collision Risk Index (CRI), Emergency Stop Frequency (ESF), and Human–Robot Interaction Indicator (HRI), each described using three linguistic terms: low, medium, and high.

According to the combinatorial structure of the fuzzy rule base, the total number of rules is equal to 27, ensuring full coverage of all possible operational states. The rules were formulated based on expert knowledge and reflect the safety-critical nature of interaction-related phenomena in autonomous logistics systems (Table A15).

**Table A15:** Complete rule base for interaction-level fuzzy subsystem.

Rule	CRI	ESF	HRI	Interaction Risk *R_i_*
R1	Low	Low	Low	Low
R2	Low	Low	Medium	Low
R3	Low	Low	High	Moderate
R4	Low	Medium	Low	Low
R5	Low	Medium	Medium	Moderate
R6	Low	Medium	High	Moderate
R7	Low	High	Low	Moderate
R8	Low	High	Medium	Moderate
R9	Low	High	High	High
R10	Medium	Low	Low	Moderate
R11	Medium	Low	Medium	Moderate
R12	Medium	Low	High	Moderate
R13	Medium	Medium	Low	Moderate
R14	Medium	Medium	Medium	Moderate
R15	Medium	Medium	High	High
R16	Medium	High	Low	High
R17	Medium	High	Medium	High
R18	Medium	High	High	High
R19	High	Low	Low	High
R20	High	Low	Medium	High
R21	High	Low	High	High
R22	High	Medium	Low	High
R23	High	Medium	Medium	High
R24	High	Medium	High	High
R25	High	High	Low	High
R26	High	High	Medium	High
R27	High	High	High	High

### Appendix B.1. Interpretation of the Rule Base

The proposed rule base follows a partially non-compensatory reasoning strategy, in which safety-critical indicators (particularly the CRI) are assigned priority over performance-related indicators. In addition, the structure of the rule base reflects several important modeling assumptions:

- Dominance of the collision-related risk (CRI): when the CRI is high, the resulting interaction risk is classified as high for all possible combinations of the ESF and HRI (R19–R27);
- Escalation effect of emergency stops (ESF): increasing the ESF shifts the risk level from low/moderate toward high, particularly when combined with a medium or high CRI (R16–R18, R25–R27);
- Amplifying role of human–robot interaction (HRI): a high HRI increases the likelihood of moderate or high risk, especially when combined with a medium or high CRI (R12, R15, and R21);
- Compensation behavior: in cases where all indicators are low or only one is elevated, the resulting risk remains low or moderate (R1–R5, R10).

Although limited compensation between secondary indicators is allowed in non-critical states, no compensation is permitted when a high CRI condition is present. This ensures that collision-related hazards remain dominant within the safety-oriented decision logic. In particular, a high value for the Collision Risk Index is treated as a dominant hazard indicator and leads to a high interaction risk in nearly all rule combinations. This reflects the underlying safety philosophy that imminent collision hazards should not be neutralized by favorable values of less critical operational indicators.

### Appendix B.2. Remarks for Reproducibility

The presented rule base is complete and can be directly implemented in a Mamdani fuzzy inference system. Together with membership functions (Section 4.3), KPI normalization (Section 5.5.1), and weights from fuzzy AHP (Section 5.4), it enables full reproducibility of the interaction-level risk evaluation.

## Appendix C. Detailed Fuzzy Inference Calculations for Subsystem Risk Evaluation

Appendix C provides the complete numerical implementation of the Mamdani fuzzy inference process used in the case study. For each subsystem (robot level, interaction level and system level), the appendix presents: (i) the normalized KPI values, (ii) the calculated membership degrees, (iii) all activated fuzzy rules together with their firing strengths, (iv) the aggregated output membership function obtained using the maximum aggregation operator, and (v) the numerical centroid defuzzification resulting in the final subsystem risk indicator. These calculations enable full reproducibility of the reported results.

The aggregated output membership functions and centroid values were obtained directly from the implemented Mamdani fuzzy inference systems in MATLAB R2024, using the *evalfis* function with centroid defuzzification.

### Appendix C.1. Robot-Level Risk Evaluation

The robot-level fuzzy subsystem includes four input indicators: the Condition Monitoring Index (CMI), Mean Time Between Failures (MTBF), Navigation Reliability (NR), and Task Completion Rate (TCR). Each input variable is described using three triangular membership functions (low, medium, and high). For the analyzed operating condition, the normalized input vector was:

[CMI, MTBF, NR, TCR] = [0.72, 0.62, 0.92, 0.96]

The obtained membership degrees are in Table A16.

**Table A16:** Obtained membership degrees for *R_r_.*

Indicator	Low	Medium	High
CMI	0.000	0.267	0.300
MTBF	0.000	0.600	0.050
NR	0.000	0.000	0.800
TCR	0.000	0.000	0.900

Due to overlapping membership functions, four fuzzy rules were activated. The corresponding firing strengths were calculated using the minimum operator (Table A17).

**Table A17:** Summary of fuzzy subsystem evaluation for *R_r_.*

Rule	Rule Antecedent	Output	Firing Strength
R77	CMI = Medium AND MTBF = Medium AND NR = High AND TCR = High	Low	0.267
R78	CMI = High AND MTBF = Medium AND NR = High AND TCR = High	Low	0.300
R80	CMI = Medium AND MTBF = High AND NR = High AND TCR = High	Low	0.050
R81	CMI = High AND MTBF = High AND NR = High AND TCR = High	Low	0.050

The truncated output membership functions were aggregated using the maximum operator. The centroid defuzzification method resulted in *R_r_* = 0.1692, which corresponds to a low robot-level risk state.

### Appendix C.2. Interaction-Level Risk Evaluation

The robot-level fuzzy subsystem includes four input indicators: the Condition Monitoring Index (CMI), Mean Time Between Failures (MTBF), Navigation Reliability (NR), and Task Completion Rate (TCR). Each input variable is described using three triangular membership functions (low, medium, and high). For the analyzed operating condition, the normalized input vector was:

[CRI, ESF, HRI] = [0.62, 0.91, 0.59]

The fuzzification results are given in Table A18.

**Table A18:** Obtained membership degrees for *R_i_.*

Indicator	Low	Medium	High
CRI	0.000	0.600	0.050
ESF	0.000	0.000	0.775
HRI	0.000	0.700	0.000

Two rules from the 27-rule interaction-level rule base were activated (according to Table A19).

**Table A19:** Summary of fuzzy subsystem evaluation for *R_i_.*

Rule	Rule Antecedent	Output	Firing Strength
R17	CRI = Medium AND ESF = High AND HRI = Medium	High	0.600
R26	CRI = High AND ESF = High AND HRI = Medium	High	0.050

After aggregation and centroid defuzzification, *R_i_* = 0.8541. The obtained value corresponds to a high interaction-level risk state. The result is mainly associated with the high membership degree of the ESF and its influence on the activated rules.

### Appendix C.3. System-Level Risk Evaluation

The system-level fuzzy subsystem consists of two input indicators: the Congestion Index (CI) and System Coordination Reliability (SCR). The normalized input vector was:

[CI, SCR] = [0.55, 0.89]

The membership degrees are given in Table A20.

**Table A20:** Obtained membership degrees for *R_s_.*

Indicator	Low	Medium	High
CI	0.000	0.833	0.000
SCR	0.000	0.000	0.725

Only one rule from the nine-rule system-level rule base was activated (according to Table A21).

**Table A21:** Summary of fuzzy subsystem evaluation for *R_s_*

Rule	Rule Antecedent	Output	Firing Strength
R6	CI = Medium AND SCR = High	Low	0.725

The aggregated output fuzzy set was defuzzified using the centroid method, with *R_s_* = 0.1380. The obtained value indicates a low system-level risk state.

### Appendix C.4. Summary of Subsystem Risk Outputs

Table A22 summarizes the complete fuzzy inference results.

**Table A22:** Summary of fuzzy subsystem evaluation.

Subsystem	Activated Rules	Maximum Firing Strength	Defuzzified Risk Output
Robot-level	R77, R78, R80, R81	0.300	0.1692
Interaction-level	R17, R26	0.600	0.8541
System-level	R6	0.725	0.1380

The results confirm that the final subsystem risk indicators are obtained from the complete Mamdani inference procedure, including fuzzification, rule activation, aggregation, and centroid defuzzification.

## References

[1] Fragapane G., de Koster R., Sgarbossa F., Strandhagen J.O. (2021). Planning and Control of Autonomous Mobile Robots for Intralogistics: Literature Review and Research Agenda. Eur. J. Oper. Res..

[2] Efthymiou O.K., Ponis S.T. (2021). Industry 4.0 Technologies and Their Impact in Contemporary Logistics: A Systematic Literature Review. Sustainability.

[3] Lemstra M.A.M.S., de Mesquita M.A. (2023). Industry 4.0: A Tertiary Literature Review. Technol. Forecast. Soc. Change.

[4] Le-Anh T., De Koster M.B.M. (2006). A Review of Design and Control of Automated Guided Vehicle Systems. Eur. J. Oper. Res..

[5] Unger H., Markert T., Müller E. (2018). Evaluation of Use Cases of Autonomous Mobile Robots in Factory Environments. Procedia Manuf..

[6] da Costa Barros Í.R., Nascimento T.P. (2021). Robotic Mobile Fulfillment Systems: A Survey on Recent Developments and Research Opportunities. Rob. Auton. Syst..

[7] Hollnagel E. (2014). From Safety-I to Safety-II: A Brief Introduction to Resilience Engineering. J. Oper. Res. Soc. Jpn..

[8] Giel R., Dąbrowska A. (2022). Autonomous Mobile Robots (AMRs) Implementation Tool Based on Simulation Model in the Context of Logistics 4.0. Proceedings of the CLC 2022 Conference Proceedings.

[9] Belzile B., Wanang-Siyapdjie T., Karimi S., Braga R.G., Iordanova I., St-Onge D. (2025). From Safety Standards to Safe Operation with Mobile Robotic Systems Deployment. arXiv.

[10] Lackner T., Hermann J., Kuhn C., Palm D. (2024). Review of Autonomous Mobile Robots in Intralogistics: State-of-the-Art, Limitations and Research Gaps. Procedia CIRP.

[11] Liu Y., Wang S., Xie Y., Xiong T., Wu M. (2024). A Review of Sensing Technologies for Indoor Autonomous Mobile Robots. Sensors.

[12] Lewczuk K., Żuchowicz P. (2024). Virtual Reality Application for the Safety Improvement of Intralogistics Systems. Sustainability.

[13] Carlson J., Murphy R.R., Nelson A. (2004). Follow-up Analysis of Mobile Robot Failures. IEEE International Conference on Robotics and Automation.

[14] Cebollada S., Payá L., Flores M., Peidró A., Reinoso O. (2021). A State-of-the-Art Review on Mobile Robotics Tasks Using Artificial Intelligence and Visual Data. Expert Syst. Appl..

[15] Markis A., Papa M., Kaselautzke D., Rathmair M., Sattinger V., Brandstötter M. Safety of Mobile Robot Systems in Industrial Applications. Proceedings of the ARW & OAGM Workshop.

[16] Zio E., Miqueles L. (2024). Digital Twins in Safety Analysis, Risk Assessment and Emergency Management. Reliab. Eng. Syst. Saf..

[17] Werbińska-Wojciechowska S., Winiarska K. (2023). Maintenance Performance in the Age of Industry 4.0: A Bibliometric Performance Analysis and a Systematic Literature Review. Sensors.

[18] Pascu I.G., Neacsu G.C., Nitu E.L., Gavriluta A.C. (2021). A Brief Review of the Methods and Techniques Used in the Innovative Internal Logistics Processes and Systems. IOP Conf. Ser. Mater. Sci. Eng..

[19] Franko Uhernik I. (2021). The Impact of Internal Logistics on the Performance of Manufacturing Companies. Chall. Futur..

[20] Saleh S.A.M., Suandi S.A., Ibrahim H., Hamad Q.S., Al Amoudi I., Ahmad N.S., Mohamad-Saleh J., Teh J. (2024). AGVs and AMRs Robots: A Brief Overview of the Differences and Navigation Principles. International Conference on Robotics, Vision, Signal Processing and Power Applications.

[21] Oyekanlu E.A., Smith A.C., Thomas W.P., Mulroy G., Hitesh D., Ramsey M., Kuhn D.J., McGhinnis J.D., Buonavita S.C., Looper N.A. (2020). A Review of Recent Advances in Automated Guided Vehicle Technologies: Integration Challenges and Research Areas for 5G-Based Smart Manufacturing Applications. IEEE Access.

[22] Chinmay K.S., Nisha G.K. (2024). Review of Autonomous Mobile Robots. 2024 International Conference on E-mobility, Power Control and Smart Systems (ICEMPS).

[23] Sharma N., Pandey J.K., Mondal S. (2023). A Review of Mobile Robots: Applications and Future Prospect. Int. J. Precis. Eng. Manuf..

[24] Keith R., La H.M. (2024). Review of Autonomous Mobile Robots for the Warehouse Environment. arXiv.

[25] Stancliff S., Dolan J., Trebi-Ollennu A. (2005). Towards a Predictive Model of Mobile Robot Reliability.

[26] Astarita V., Guido G., Haghshenas S.S., Haghshenas S.S. (2024). Risk Reduction in Transportation Systems: The Role of Digital Twins According to a Bibliometric-Based Literature Review. Sustainability.

[27] Wuennenberg M., Muehlbauer K., Fottner J., Meissner S. (2023). Towards Predictive Analytics in Internal Logistics—An Approach for the Data-Driven Determination of Key Performance Indicators. CIRP J. Manuf. Sci. Technol..

[28] Loganathan A., Ahmad N.S. (2023). A Systematic Review on Recent Advances in Autonomous Mobile Robot Navigation. Eng. Sci. Technol. Int. J..

[29] Waga A., Benhlima S., Bekri A., Abdouni J., Saber F.Z. (2025). A Survey on Autonomous Navigation for Mobile Robots: From Traditional Techniques to Deep Learning and Large Language Models. J. King Saud Univ.-Comput. Inf. Sci..

[30] Alenjareghi M.J., Keivanpour S., Chinniah Y.A., Jocelyn S., Oulmane A. (2024). Safe Human-Robot Collaboration: A Systematic Review of Risk Assessment Methods with AI Integration and Standardization Considerations.

[31] Kumar S.D., R A.R. (2024). A Study of Risk Management Practices in Logistics Operations. Int. J. Lit. Educ..

[32] (2018). Risk Management—Guidelines.

[33] Bekishev Y., Pisarenko Z., Arkadiev V. (2023). FMEA Model in Risk Analysis for the Implementation of AGV/AMR Robotic Technologies into the Internal Supply System of Enterprises. Risks.

[34] Liu H.C., Liu L., Bian Q.H., Lin Q.L., Dong N., Xu P.C. (2011). Failure Mode and Effects Analysis Using Fuzzy Evidential Reasoning Approach and Grey Theory. Expert Syst. Appl..

[35] Ruijters E., Stoelinga M. (2015). Fault Tree Analysis: A Survey of the State-of-the-Art in Modeling, Analysis and Tools. Comput. Sci. Rev..

[36] Cai B., Kong X., Liu Y., Lin J., Yuan X., Xu H., Ji R. (2019). Application of Bayesian Networks in Reliability Evaluation. IEEE Trans. Ind. Inform..

[37] Pandit P., Earthperson A., Diaconeasa M.A. (2025). The Development, Implementation, and Application of a Probabilistic Risk Assessment Framework to Evaluate Supply Chain Shortages. Logistics.

[38] Ustundag A., Greco S., Bouchon-Meunier B., Coletti G., Fedrizzi M., Matarazzo B., Yager R.R. (2012). Risk Evaluation of Warehouse Operations Using Fuzzy FMEA. Advances in Computational Intelligence. IPMU 2012. Communications in Computer and Information Science.

[39] Zheng X., Liu Q., Li Y., Wang B., Qin W. (2025). Safety Risk Assessment for Connected and Automated Vehicles: Integrating FTA and CM-Improved AHP. Reliab. Eng. Syst. Saf..

[40] Markowski A.S., Mannan M.S., Bigoszewska A. (2009). Fuzzy Logic for Process Safety Analysis. J. Loss Prev. Process Ind..

[41] Morse J., Araiza-Illan D., Eder K., Lawry J., Richards A. (2017). A Fuzzy Approach to Qualification in Design Exploration for Autonomous Robots and Systems. 2017 IEEE International Conference on Fuzzy Systems (FUZZ-IEEE).

[42] Aslantas M., Kutlu Gündoğdu F., Moslem S. (2025). Evaluating the Potential Risks Posed by Autonomous Vehicles by Using a Decomposed Fuzzy Multi-Criteria Decision-Making Model. Transp. Eng..

[43] Merola F., Bernardeschi C., Lami G. (2024). A Risk Assessment Framework Based on Fuzzy Logic for Automotive Systems. Safety.

[44] Díaz-Curbelo A., Gento Á.M., Redondo A., Aqlan F. (2019). A Fuzzy-Based Holistic Approach for Supply Chain Risk Assessment and Aggregation Considering Risk Interdependencies. Appl. Sci..

[45] Siami M., Naderpour M., Ramezani F., Lu J. (2024). Risk Assessment Through Big Data: An Autonomous Fuzzy Decision Support System. IEEE Trans. Intell. Transp. Syst..

[46] Mahmood A., Szabolcsi R. (2025). A Systematic Review on Risk Management and Enhancing Reliability in Autonomous Vehicles. Machines.

[47] Zuhr P., Rissmann L., Meißner S. (2022). Framework for Planning and Implementation of Digital Process Twins in the Field of Internal Logistics. IFAC-PapersOnLine.

[48] Zhu M.-L. (2023). Digital Twin for Integration of Design, Manufacturing, and Maintenance. Res. Outreach.

[49] Kritzinger W., Karner M., Traar G., Henjes J., Sihn W. (2018). Digital Twin in Manufacturing: A Categorical Literature Review and Classification. IFAC-PapersOnLine.

[50] Sharma A., Kosasih E., Zhang J., Brintrup A., Calinescu A. (2022). Digital Twins: State of the Art Theory and Practice, Challenges, and Open Research Questions. J. Ind. Inf. Integr..

[51] Hananto A.L., Tirta A., Herawan S.G., Idris M., Soudagar M.E.M., Djamari D.W., Veza I. (2024). Digital Twin and 3D Digital Twin: Concepts, Applications, and Challenges in Industry 4.0 for Digital Twin. Computers.

[52] Lin K.Y., Yao Y. (2022). A Review of Digital Twin in Logistics: Applications and Future Works. J. Eng. Manag. Syst. Eng..

[53] Ashrafian A., Pedersen S. (2023). Digital Twin for Complex Logistics Systems: The Case Study of a Large-Scale Automated Order Picking and Fulfillment System. IFAC-PapersOnLine.

[54] Leng J., Yan D., Liu Q., Zhang H., Zhao G., Wei L., Zhang D., Yu A., Chen X. (2021). Digital Twin-Driven Joint Optimisation of Packing and Storage Assignment in Large-Scale Automated High-Rise Warehouse Product-Service System. Int. J. Comput. Integr. Manuf..

[55] Errandonea I., Beltrán S., Arrizabalaga S. (2020). Digital Twin for Maintenance: A Literature Review. Comput. Ind..

[56] Werbińska-Wojciechowska S., Giel R., Winiarska K. (2024). Digital Twin Applications in Internal Transportation Systems Maintenance: A Conceptual Framework. Proceedings of the 2024 8th International Conference on System Reliability and Safety, ICSRS 2024.

[57] Vashishth T.K., Sharma V., Sharma K.K., Kumar B., Chaudhary S., Panwar R., Iyer S., Nayyar A., Paul A., Naved M. (2025). Chapter 16—Digital Twins Solutions for Smart Logistics and Transportation. Digital Twins for Smart Cities and Villages.

[58] Negri E., Fumagalli L., Macchi M. (2017). A Review of the Roles of Digital Twin in CPS-Based Production Systems. Procedia Manuf..

[59] Li S., Jin P., Su S., Yao J., Pang Q. (2025). Digital Twins and Cross-Border Logistics Systems Risk Management Capability: An Innovation Diffusion Perspective. Systems.

[60] Zaidi S.A.H., Khan S.A., Chaabane A. (2024). Unlocking the Potential of Digital Twins in Supply Chains: A Systematic Review. Supply Chain Anal..

[61] Li J., Wang J. (2025). Digital Twin-Driven Management Strategies for Logistics Transportation Systems. Sci. Rep..

[62] Eko Virando G., Lee B., Kee S.H., Yee J.J. (2024). Digital Twin Simulation and Implementation in Safety Risk Management Process. IEEE Access.

[63] Kampourakis K.E., Gkioulos V., Kavallieratos G., Lin J.C. (2025). Digital Twin-Enabled Incident Detection and Response: A Systematic Review of Critical Infrastructures Applications. Int. J. Inf. Secur..

[64] Sajadieh S.M.M., Noh S. (2025). Do From Simulation to Autonomy: Reviews of the Integration of Artificial Intelligence and Digital Twins.

[65] Alam M.M., Ugli R.A.N., Tanha K.J., Jun T. (2026). AI-Enhanced Digital Twin Systems for Warehouse Logistics Optimization: A Review of Challenges with Solutions and Future Directions. ICT Express.

[66] Mohanraj R., Vaishnavi B.K. (2025). Data Enabling Technology in Digital Twin and Its Frameworks in Different Industrial Applications. J. Ind. Inf. Integr..

[67] Okimi T.O., Adeoye O., Omoboye O. (2026). Integrating Digital Twins and Decision Support Systems for Predictive Risk Management in Urban Renewal. Digit. Twin.

[68] Galkin A., Samchuk G., Kopytkov D., Thompson R.G. (2025). Digital Twins in Logistics: A Comprehensive Bibliometric Analysis for Advancing Smart Cities and Sustainable Development. Discov. Sustain..

[69] Berti N., Serena F. (2022). Digital Twin and Human Factors in Manufacturing and Logistics Systems: State of the Art and Future Research Directions. IFAC-PapersOnLine.

[70] You Y., Chen C., Hu F., Liu Y., Ji Z. (2022). Advances of Digital Twins for Predictive Maintenance. Procedia Comput. Sci..

[71] Mahmoodian M., Shahrivar F., Setunge S., Mazaheri S. (2022). Development of Digital Twin for Intelligent Maintenance of Civil Infrastructure. Sustainability.

[72] Zhang L., Guo J., Fu X., Tiong R.L.K., Zhang P. (2024). Digital Twin Enabled Real-Time Advanced Control of TBM Operation Using Deep Learning Methods. Autom. Constr..

[73] (2020). Automation Systems and Integration—Digital Twin Framework for Manufacturing—Part 1: Overview and General Principles.

[74] (2021). Automation Systems and Integration—Digital Twin Framework for Manufacturing—Part 2: Reference Architecture.

[75] (2020). Automation Systems and Integration—Digital Twin Framework for Manufacturing—Part 3: Digital Representation of Manufacturing Elements.

[76] (2021). Automation Systems and Integration—Digital Twin Framework for Manufacturing—Part 4: Information Exchange.

[77] Kubler S., Robert J., Derigent W., Voisin A., Le Traon Y. (2016). A State-of the-Art Survey & Testbed of Fuzzy AHP (FAHP) Applications. Expert Syst. Appl..

[78] Leung L.C., Cao D. (2000). On Consistency and Ranking of Alternatives in Fuzzy AHP. Eur. J. Oper. Res..

[79] Dongre J.K., Raundale A.R. (2023). Examining the Fuzzy Inference on Mamdani Fuzzy Inference System and Takagi-Sugeno Fuzzy Model. J. Harbin Eng. Univ..

